# Alginate and chitosan-coated ferulic acid-loaded selenium nanoparticles: synthesis, characterization, and anticancer activity against MDA-MB-231 breast cancer cells

**DOI:** 10.1007/s12032-025-02756-8

**Published:** 2025-05-05

**Authors:** Duygu Petunya Çetin, Mücahit Seçme, Hasan İlhan, Necdet Sağlam

**Affiliations:** 1 Department of Biotechnology, Institute of Biotechnology, Ankara University, Ankara, Turkey; 2 Department of Medical Biology, Faculty of Medicine, Ordu University, Ordu, Turkey; 3 Department of Nanotechnology and Nanomedicine, Graduate School of Science and Engineering, Hacettepe University, Ankara, Turkey

**Keywords:** Breast carcinoma, Triple-negative, Breast cancer, MDA-MB-231 cells, Selenium nanoparticles, Alginate, Chitosan, Ferulic acid

## Abstract

Triple-negative breast cancer (TNBC), characterized by its aggressive behavior and lack of targeted therapies, remains a major therapeutic challenge. This study presents the synthesis and evaluation of ferulic acid-loaded selenium nanoparticles (FA-SeNPs) coated with alginate (Alg@FA-SeNPs) and chitosan (CS@FA-SeNPs) as potential nanocarriers for TNBC treatment. Ferulic acid was selected for its pro-apoptotic and anti-metastatic properties, despite its limited bioavailability. Encapsulation in SeNPs enhanced its stability and delivery efficiency. Alg@FA-SeNPs exhibited greater cytotoxicity (IC50: 103.6 µg/mL) than CS@FA-SeNPs (IC50: 178 µg/mL) after 48 h. Gene expression analyses showed significant H2AX upregulation with Alg@FA-SeNPs, indicating genotoxic stress, and marked Bcl-2 downregulation with CS@FA-SeNPs, favoring apoptosis. Zeta potential measurements confirmed near-neutral surface charge for Alg@FA-SeNPs and strong positive charge for CS@FA-SeNPs, supporting good colloidal stability. These findings highlight the therapeutic promise of biopolymer-coated SeNPs, particularly alginate-coated formulations, as targeted drug delivery systems for TNBC.

**Supplementary Information:**

The online version contains supplementary material available at 10.1007/s12032-025-02756-8.

## Introduction

Cancer has become a leading cause of death with an increasing number of cases globally [1, 2]. Among the 20 million new cancer cases and 9.7 million deaths reported by GLOBOCAN 2022, female breast cancer accounted for 11.6% of all cases and 6.9% of deaths [1]. These data show that female breast cancer is one of the most common cancer types in terms of both incidence and mortality [1]. The MDA-MB-231 cell line is a model of triple-negative breast cancer (TNBC), one of the subtypes of breast cancer, and has an aggressive, invasive, and poorly differentiated nature [3]. TNBC is characterized by lacking estrogen receptor (ER), progesterone receptor (PR), and human epidermal growth factor receptor 2 (HER2) expression. TNBC accounts for approximately 15–20% of all breast cancer cases. This subtype has a poor prognosis relative to other subtypes [4, 5]. MDA-MB-231 cells secrete high levels of matrix metalloproteinases (MMPs) that promote invasive behavior by facilitating the breakdown of extracellular matrix [6]. In addition, these cells carry a p53 mutation that allows them to avoid apoptotic conditions and a KRas mutation that promotes uncontrolled cell proliferation [7, 8]. Due to the lack of ER, PR, and HER2, MDA-MB-231 cells do not respond to receptor-targeting hormonal therapies, such as tamoxifen, or HER2-targeted therapies, such as trastuzumab, posing significant therapeutic challenges [9].

In recent years, nanoparticles have been widely used as drug delivery systems in cancer research. The limitations of currently used conventional drugs, such as systemic toxicity, high dose requirements, and resistance to treatment, have led to the need to develop nanotechnology-based strategies [10]. Nanoparticles offer many advantages such as enhanced bioavailability, targeted delivery, and controlled drug release. Their small size facilitates cellular uptake and enables efficient delivery of therapeutic agents to tumor sites [11]. Furthermore, the enhanced permeability and retention (EPR) effect allows nanoparticles to passively accumulate in tumor tissues due to the leaky vascular structure and impaired lymphatic drainage in tumor sites [12].

Selenium, an essential trace element, plays an important role in various physiological processes, including DNA synthesis, thyroid hormone metabolism, immune function, and protection against oxidative stress. Selenium functions as a redox center in antioxidant enzymes, such as glutathione peroxidase, which contributes to the breakdown of peroxides [13]. Selenium also exhibits antimicrobial properties by oxidizing intracellular thiols [14]. Compared to conventional forms of selenium, selenium nanoparticles (SeNPs) have higher bioavailability and absorption. SeNPs have attracted considerable interest as nanocarrier systems due to their unique physicochemical properties, biocompatibility, and anticancer potential [15, 16].

Selenium nanoparticles (SeNPs) have attracted considerable interest due to their anticancer potential, a finding increasingly supported by scientific evidence. Notably, these nanoparticles are capable of inducing apoptosis in diverse cancer cell lines, such as those originating from breast, prostate, and hepatocellular carcinomas [17]. Among the various ways selenium nanoparticles combat cancer is their significant impact on calcium signaling and their ability to trigger endoplasmic reticulum (ER) stress, both critical factors in promoting apoptosis within cancer cells [17]. SeNPs have demonstrated the ability to overcome multidrug resistance in cancer therapy. They can inhibit the function of multidrug efflux pumps and enhance the cytotoxic activity of chemotherapeutic drugs, making them effective against drug-resistant cancer cells [18]. SeNPs exhibit low toxicity to normal cells while being highly cytotoxic to cancer cells. This selective cytotoxicity is a significant advantage, as it reduces the side effects typically associated with conventional cancer therapies [19].

Ferulic acid (FA), a naturally occurring hydroxycinnamic acid, is found in plant cell walls [20]. It exhibits anticancer effects through various mechanisms, such as induction of apoptosis, modulation of oxidative stress, inhibition of angiogenesis, and suppression of metastasis. Ferulic acid triggers apoptosis and autophagic cell death by altering the expression of *procaspase-3, Bcl-2*, and *Bax* and specifically regulates the MAPK pathway through phosphorylation of ERK and JNK [21]. Ferulic acid inhibits angiogenesis by targeting the fibroblast growth factor receptor 1 (FGFR1) and blocking the PI3K-Akt signaling pathway, which is critical for tumor growth and vascularization [22]. In breast cancer, it suppresses metastasis by reversing the epithelial-mesenchymal transition (EMT), thereby reducing tumor cell viability and metastatic potential [23]. Despite its promising anticancer properties, the clinical application of ferulic acid remains limited due to its poor solubility, rapid metabolism, and low bioavailability [24]. Encapsulation of ferulic acid in nanocarriers offers a viable strategy to overcome these limitations by enhancing its stability, bioavailability, and therapeutic efficacy.

The technique of encapsulating nanoparticles with biopolymers can be used to increase the effectiveness of medication delivery systems based on nanoparticles and to keep them stable until they arrive at their destination. Natural polymers such as alginate and chitosan are widely used as nanoparticle coating materials [25]. These molecules enhance the biological compatibility, stability, and targeting capacity of the delivery systems. Alginate (Alg), a biodegradable polysaccharide derived from seaweed, is an attractive option in biomedical applications due to its high biocompatibility, non-toxicity, and gelling ability [25, 26]. Alginate’s structure allows it to create an environment that is suitable for controlled drug release. Chitosan (CS), a biopolymer derived from chitin, is a biocompatible and biodegradable material [25, 26]. With antimicrobial properties, chitosan has a high mucoadhesion capacity that facilitates the adhesion of nanoparticles to mucosal membranes [25, 26]. Moreover, the abundance of reactive amino groups along the polymer backbone of chitosan is one of the reasons why it is frequently used in nano drug delivery systems. Through protonation in acidic conditions, the amino groups increase the solubility of chitosan in such environments [2]. Its positively charged structure facilitates interactions with negatively charged biomolecules, while its pH sensitivity enables efficient drug transport and release in various environments [25, 26].

This study investigates the use of selenium nanoparticles (SeNPs) as nanocarriers for the delivery of ferulic acid to improve its anticancer efficacy, against MDA-MB-231 breast cancer cells. Ferulic acid-loaded Selenium Nanoparticles (FA-SeNPs) were coated with alginate (Alg@FA-SeNPs) and chitosan (CS@FA-SeNPs) to enhance their stability, biocompatibility, and drug release characteristics. The physicochemical properties of the coated nanoparticles, including size, structure, stability, and release profiles, were thoroughly characterized. Furthermore, their anticancer potential was evaluated using MDA-MB-231 cells to determine their effectiveness in inhibiting cancer cell proliferation and promoting cell death. This research highlights the potential of SeNPs coated with different biopolymers as an innovative drug delivery platform for ferulic acid, contributing to the development of advanced therapeutic strategies for breast cancer treatment.

## Materials and methods

### Preparation of ferulic acid-loaded selenium nanoparticles (FA-SeNPs)

Selenium nanoparticles (SeNPs) were synthesized via the reduction of sodium selenite using ascorbic acid, with Tween 20 employed as a stabilizing agent. Specifically, 2 mL of sodium selenite (100 μM) and 2 mL of Tween 20 were dissolved in 18 mL of distilled water, and the resulting solution was placed on a magnetic stirrer at 200 rpm. Separately, 0.1 g of ferulic acid was dissolved in 10 mL of dimethylformamide (DMF) and subsequently added to the selenium solution. The mixture was stirred on a magnetic stirrer until it achieved clarity. Following this, 1 mL of ascorbic acid (240 mM) was introduced dropwise into the solution. The final mixture was incubated on a magnetic stirrer at 265 rpm in the dark for 4 h. The synthesized SeNP solution was then dialyzed for 24 h to remove any unreacted components [27].

### Preparation of alginate-coated ferulic acid-loaded selenium nanoparticles (Alg@FA-SeNPs)

A 0.06% alginate solution was prepared by dissolving sodium alginate in 100 mL of distilled water, followed by incubation on a magnetic stirrer at 300 rpm until the solution became transparent. An 18 mM CaCl₂ solution was prepared separately. Selenium nanoparticles (SeNPs), previously synthesized, were mixed with the alginate solution at a 4:1 ratio and subjected to sonication for 5 min. The mixture was subsequently incubated on a magnetic stirrer at 300 rpm for 30 min. To facilitate cross-linking, 500 μL of the prepared CaCl₂ solution was added dropwise to the SeNP-alginate solution and incubated for 1 h. The resultant nanoparticles were collected by centrifugation at 14,000 rpm for 20 min then resuspended with distilled water and stored at room temperature for further use [28, 29].

### Preparation of chitosan-coated ferulic acid-loaded selenium nanoparticles (CS@FA-SeNPs)

To prepare CS-NP, ionic gelation of chitosan with tripolyphosphate pentasodium (TPP) anions is utilized/employed. Chitosan is dissolved at a concentration of 0.2% (w/v) in acetic acid (1% v/v) and stirred at 200 rpm overnight. Selenium nanoparticles (SeNPs), previously synthesized, were mixed with the chitosan solution at a 1:1 ratio. TPP is dissolved at 0.5% (w/v) in ultrapure water. For cross-linking, chitosan and TPP solutions are mixed in a 1:1:1 volume ratio under magnetic stirring at 270 rpm. The resulting formulation is centrifuged at 14,000 rpm for 15 min, and the pellet is resuspended in phosphate-buffered saline (PBS) and stored at room temperature for further use [30].

### Determination of drug release profiles of Alg@FA-SeNPs and CS@FA-SeNPs

To evaluate drug release from the nanoparticles, the nanoparticles were first collected via centrifugation. Acetate buffer (pH 5.5) and PBS (pH 7.4) were added onto pellet, respectively, to simulate different release environments. The resuspended nanoparticles were then incubated at either 27 or 37 °C with gentle agitation at 90 rpm to assess temperature-dependent release behavior. At different time intervals, 100 μL of the release medium was taken for analysis, and an equal volume of fresh buffer was added. The drug release was quantified by measuring the absorbance of the collected release media at 320 nm using a TECAN Infinite® M Plex, multimode microplate reader. Each experimental group was analyzed in triplicate, consisting of three biological replicates and three technical replicates per batch, to ensure statistical robustness and reproducibility.

### Determination of encapsulation efficiency

The encapsulation efficiency (EE) and drug loading capacity (DLC) of the nanoparticles were determined using a dialysis-based method. A defined volume of the nanoparticle suspension was transferred into a dialysis bag (molecular weight cut-off: 2 kD) and dialyzed against 800 mL of distilled water under continuous stirring at 250 rpm at room temperature for 24 h to allow the diffusion of the unencapsulated (free) drug. After dialysis, the dialysis water was collected, and the concentration of free drug was determined spectrophotometrically at 320 nm using a UV–Vis spectrophotometer (TECAN Infinite® M Plex multimode microplate reader). Drug concentration was calculated based on a previously prepared standard calibration curve.

The amount of drug encapsulated within the nanoparticles was calculated by subtracting the amount of free drug in the supernatant from the initial amount of drug used in the formulation. The encapsulation efficiency (EE%) and drug loading capacity (DLC%) were calculated using the following equations:

$$ {\text{EE }}\left( {\text{\% }} \right){ } = { }\frac{{{\text{mass }}\,{\text{of}}\,{\text{total}}\,{\text{drug}}\,{\text{added}}\,\left( {{\text{mg}}} \right) - {\text{mass }}\,{\text{of}}\,{\text{free}}\,{\text{drug}}\,\left( {{\text{mg}}} \right)}}{{{\text{mass}}\,{\text{ of}}\,{\text{total}}\,{\text{drug}}\,{\text{added}}\,\left( {{\text{mg}}} \right)}} \times 100 $$

### Characterization of nanoparticles

The synthesized solutions of FA-SeNPs, Alg@FA-SeNPs, and CS@FA-SeNPs were characterized by using microscopic and spectroscopic methods. UV–vis absorbance of nanoparticles was characterized by a scanning spectrophotometer, TECAN Infinite® M Plex, multimode microplate reader. Size, structure, and elemental analysis of the nanoparticles were determined by FIB-SEM equipped with EDX using TESCAN GAIA3+ Oxford XMax 150 EDS (Hünitek Hacettepe, Turkey). Additionally, the crystalline structure of the nanoparticles was analyzed using X-ray diffraction (XRD) with a Malvern Panalytical Aeris diffractometer (Hünitek, Hacettepe, Turkey) to confirm the phase composition and assess the degree of crystallinity. The size and morphology of the nanoparticles were confirmed using Scanning electron microscope (SEM) to examine the morphology and size distribution of the nanoparticles at the nanoscale level. The size analysis from SEM images was further quantified using ImageJ, a widely used software for measuring particle size and distribution.

### Cell culture

The MDA-MB-231 triple-negative breast cancer cells were cultivated in RPMI-1640 basal medium (Gibco) with the addition of 10% fetal bovine serum (FBS) (Gibco), 20 units/mL penicillin, 20 μg/mL streptomycin, and 0.1 mM amino acid solution (Biological Industries). The cells were cultivated at 37 °C in a humidified incubator with 5% CO_2_, in accordance with standard conditions. When the cells reached sufficient confluence under appropriate conditions, they were passaged and cultured for the relevant assays. The proliferation status and morphology of the cells were observed using an inverted microscope. Different concentrations (25, 50, 100, 200, 400 μg/mL) of Alg@FA-SeNPs and CS@FA-SeNPs were administered to the breast cancer cells in a time- and dose-dependent manner.

### MTT cell proliferation assay

The antiproliferative effects of Alg@FA-SeNPs and CS@FA-SeNPs were analyzed by MTT assay (GoldBio, USA) according to kit protocol. The MDA-MB-231 cells (3 × 10^4^ cells/well) were cultured in 96-well plates in media for 24, 48, and 72 h. Cells were treated with increasing concentrations of 25, 50, 100, 200, and 400 μg/mL of nanoparticles for 24 h. 10 μL of MTT stock solution (5 mg/mL) was added to each well, and the plates were incubated at 37 °C for 3 h. After incubation, the media were carefully removed by aspiration. Dimethyl sulfoxide (DMSO, 100 µL/well; Sigma Aldrich, USA) was used to solubilize the formazan crystals, and the plates were incubated at 37 °C for 15 min. Finally, the absorbance was read at 570 nm with a microplate reader (Biotek-EPOCH2). Since the synthesized nanoparticles are in water-soluble form in the medium, there is no need to use a vehicle control (such as DMSO, ethanol). As a control group, the comparison was made using only the group that was not treated with nanoparticles and only the group that was given medium.

The half maximum inhibitory concentration (IC_50_) of nanoparticles was determined by using GraphPad Prism (version 9.4.1). The percentage of breast cancer cell viability was determined using the following formula:

$$ {\text{Cell}}\,{\text{Viability}}\,\left( \% \right) = \,\left[ {{{\left( {{\text{Treatment}}\,{\text{group}}\,{\text{OD}}_{{{57}0}} - {\text{Blank}}\,{\text{well}}\,{\text{OD}}_{{{57}0}} } \right)} \mathord{\left/ {\vphantom {{\left( {{\text{Treatment}}\,{\text{group}}\,{\text{OD}}_{{{57}0}} - {\text{Blank}}\,{\text{well}}\,{\text{OD}}_{{{57}0}} } \right)} {\left( {{\text{Untreated}}\,{\text{group}}\,{\text{OD}}_{{{57}0}} - {\text{Blank}}\,{\text{well}}\,{\text{OD}}_{{{57}0}} } \right)}}} \right. \kern-0pt} {\left( {{\text{Untreated}}\,{\text{group}}\,{\text{OD}}_{{{57}0}} - {\text{Blank}}\,{\text{well}}\,{\text{OD}}_{{{57}0}} } \right)}}} \right] \times 100\% $$

All experiments were performed using IC_50_ dose of nanoparticles obtained from cell proliferation assays for MDA-MB-231 cells.

### Real-time PCR analysis

MDA-MB-231 cells were treated with Alg@FA-SeNPs and CS@FA-SeNPs at the IC50 dose, as determined through MTT assay or media only (control). Total RNA was isolated via TRIzol (Invitrogen), following the protocol provided by the manufacturer. NanoDrop was used to measure the amount and quality of RNA (BioSpec-nano, Shimadzu, Japan). The High Capacity cDNA Synthesis kit with Rnase Inh. (ABT, Cat No: C03-01-20, Turkey) was utilized for cDNA synthesis. Relative RNA levels were detected using the Rotor Gene 6000 Real-time PCR Thermocycler with NucleoGene qPCR SYBER-Green Master Mix. The primer sequences for the amplification of human *BAX, BCL-2, CASPASE-3, CASPASE-8, CASPASE-9, NRF2*, and *H2A histone family member X (H2AX, gammaH2ax)* are presented in Table 1 [31, 32]. The following cycle conditions were employed: Initial denaturation at 95 °C for 15 min, followed by 15 s at 95 °C and 1 min at 60 °C for a total of 40 cycles. The expression fold-change for each target was calculated by the 2^−∆∆CT^ method, with GAPDH serving as the normalization control.

**Table 1:** Reverse and forward sequences of the primers

Gene name	Forward	Reverse
*GAPDH*	GTCTCCTCTGACTTCAACAGCG	ACCACCCTGTTGCTGTAGCCAA
*CASPASE-3*	GCAGCAAACCTCAGGGAAAC	TGTCGGCATACTGTTTCAGCA
*CASPASE-9*	GGCTGTCTACGGCACAGATGGA	CTGGCTCGGGGTTACTGCCAG
*CASPASE-8*	AGAAGAGGGTCATCCTGGGAGA	TCAGGACTTCCTTCAAGGCTGC
*BCL-2*	TGCACCTGACGCCCTTCAC	AGACAGCCAGGAGAAATCAAACAG
*BAX*	TCAGGATGCGTCCACCAAGAAG	TGTGTCCACGGCGGCAATCATC
*H2AX*	CGGCAGTGCTGGAGTACCTCA	AGCTCCTCGTCGTTGCGGATG
*NRF2*	CACATCCAGTCAGAAACCAGTGG	GGAATGTCTGCGCCAAAAGCTG

### TAS–TOS and OSI measurement:

The Rel Assay commercial kits (Rel Assay Kit Diagnostics, Gaziantep, Turkey) were utilized to evaluate the total antioxidant status (TAS) and total oxidant status (TOS) in control and MDA-MB-231 cells that had been treated with Alg@FA-SeNPs and CS@FA-SeNPs, in accordance with the manufacturer’s specified protocol. The TAS and TOS values were measured using a microplate reader (BioTek, Epoch2), and the oxidative stress index (OSI) was determined by converting TAS values from mmol to μmol units and then calculating OSI according to a previously described method [31].

### Statistical analyses

The Real-time PCR data were quantified using the 2^−∆∆CT^ method, with analysis facilitated by the Gene Globe RT-PCR Analysis RT2 Profile PCR Array Data Analysis tool (Qiagen). Statistical evaluations were conducted using GraphPad Prism 9.4.1 software. All results are presented as mean ± standard deviation (S.D.) derived from at least three independent biological replicates. Mean comparisons were performed using either an Unpaired *t* test or one-way analysis of variance (ANOVA), with Dunnett’s test applied for post hoc analysis.

## Results

### Characterization of nanoparticles

**Fig. 1:**
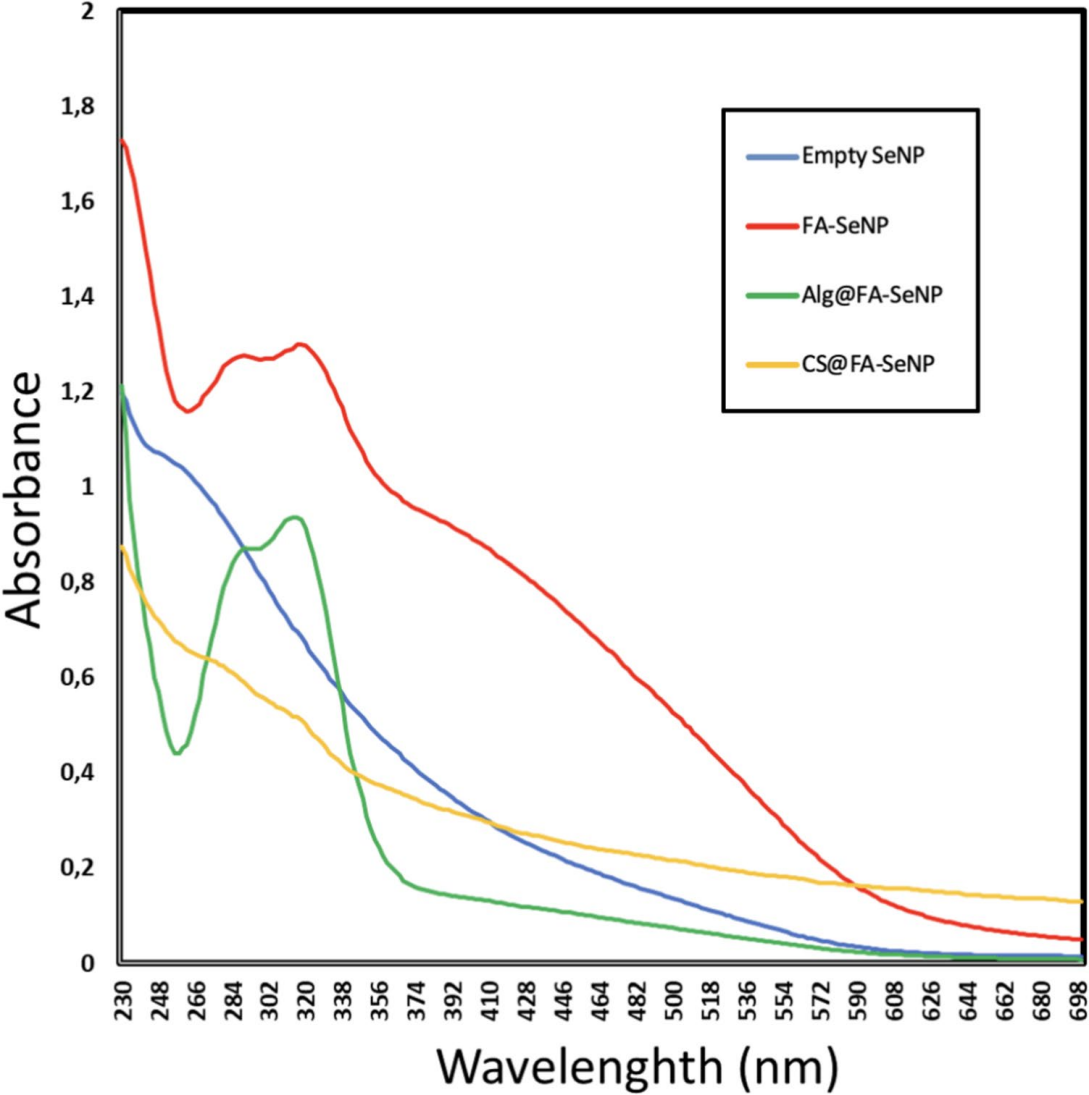
SeNPs UV–Vis absorption spectra of SeNPs, FA-SeNPs, Alg@FA-SeNPs, and CS@FA-SeNPs. The characteristic absorption peaks confirm successful loading of ferulic acid and biopolymer coatings. Alg@FA-SeNPs show a peak shift, indicating strong interactions between alginate and the nanoparticle surface, which may contribute to controlled drug release

The UV–Vis spectral shifts observed in the analysis confirm the surface chemistry changes resulting from the functionalization of SeNPs with ferulic acid, alginate, and chitosan. The unmodified selenium nanoparticles exhibited a characteristic surface plasmon resonance (SPR) peak at 266 nm, indicating their inherent optical properties. Upon loading with ferulic acid, new peaks appeared at 288 and 324 nm, corresponding to the electronic transitions of ferulic acid, confirming its successful attachment to the nanoparticle surface. The alginate-coated FA-SeNPs introduced an additional peak at 250 nm, which likely arises from the alginate structure interacting with the nanoparticle core, while the peaks at 288 and 320 nm indicated the retention of ferulic acid. The shift in peak positions suggested modified electronic interactions due to the alginate coating. Similarly, the chitosan-coated FA-SeNPs exhibited a shift in the SPR peak to 276 nm, reflecting electrostatic and hydrogen-bond interactions between chitosan and the nanoparticle surface. The presence of the 320 nm peak confirmed that ferulic acid remains attached even after chitosan coating. These spectral variations demonstrated that each surface modification introduced distinct optical changes, validating the successful functionalization of SeNPs and highlighting the influence of surface chemistry on their optical behavior (Fig. 1).

**Fig. 2:**
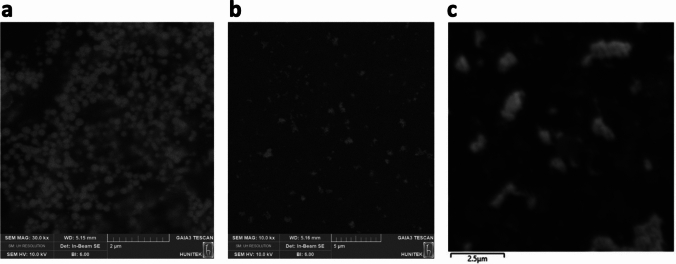
SEM images of **a** FA-SeNPs, **b** Alg@FA-SeNPs, and **c** CS@FA-SeNPs. FA-SeNPs appear uniformly distributed with a spherical morphology. Alg@FA-SeNPs show moderate aggregation, while CS@FA-SeNPs exhibit significant clustering, which may affect cellular uptake efficiency and drug release

The first SEM image shows FA-SeNPs, exhibiting uniform distribution with relatively spherical morphology and particle sizes within the nanoscale range. The second image, representing Alg@FA-SeNPs, indicated an increased level of aggregation, likely due to the alginate coating enhancing inter-particle interactions. The particle sizes appeared slightly larger compared to the uncoated SeNPs, confirming the presence of the alginate layer. The third image illustrates CS@FA-SeNPs, where significant aggregation and irregularities in morphology were observed, possibly due to the thicker and less uniform coating by chitosan. This could be attributed to the stronger binding affinity and electrostatic interactions introduced by chitosan (Fig. 2).

The particle size distribution analysis revealed (Fig. S1) distinct differences among the synthesized SeNPs. Bare SeNPs exhibited an average particle size of 119 nm, while CS@FA-SeNPs and Alg@FA-SeNPs showed increased sizes of 261 and 218 nm, respectively. The larger size of CS@FA-SeNPs can be attributed to both the presence of chitosan and its higher concentration (0.2%) compared to alginate (0.06%) in Alg@FA-SeNPs, highlighting the influence of polymer type and concentration on final particle size.

**Fig. 3:**
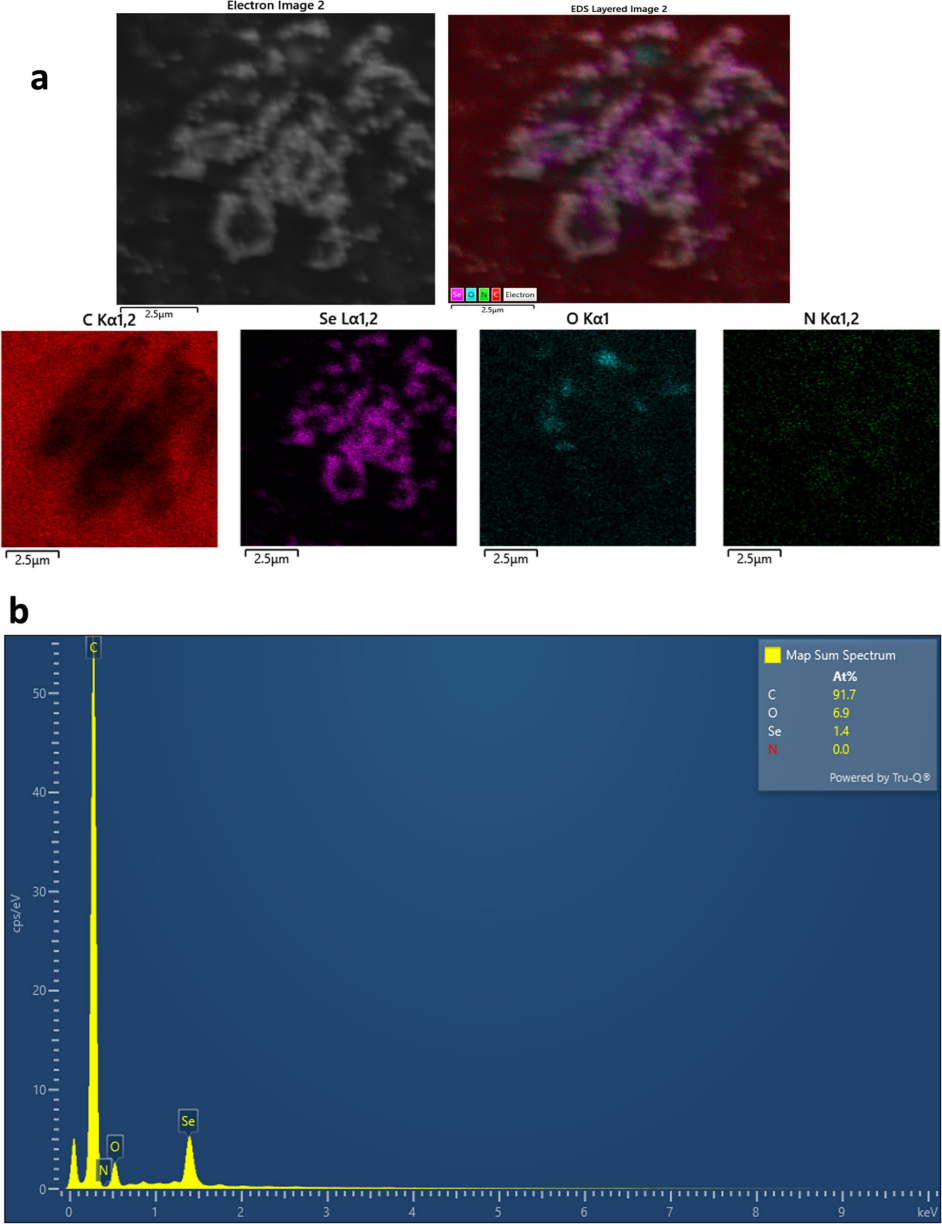
**a** SEM mapping and elemental analysis of FA-SeNPs and **b** EDX spectrum for FA-SeNPs. Selenium, oxygen, and carbon are uniformly distributed in all formulations, confirming successful synthesis

SEM-Energy-dispersive X-ray (EDX) analysis and elemental mapping were performed to confirm the successful synthesis of ferulic acid-loaded selenium nanoparticles (FA-SeNPs). The EDX spectrum revealed the presence of carbon content of 91.7 atomic percent (91.7 At%), oxygen (6.9 At%), and selenium (1.4 At%), indicating the incorporation of selenium and ferulic acid into the nanoparticle matrix. The high carbon content was attributed to the graphene coating applied to enhance the quality of SEM imaging. Elemental mapping results provided further evidence of the uniform distribution of selenium within the nanoparticles, confirming the structural integrity and successful synthesis of FA-SeNPs. These findings collectively demonstrate that the synthesis process effectively produced FA-SeNPs with consistent elemental composition and uniform distribution, meeting the goals of the study (Fig. 3).

**Fig. 4:**
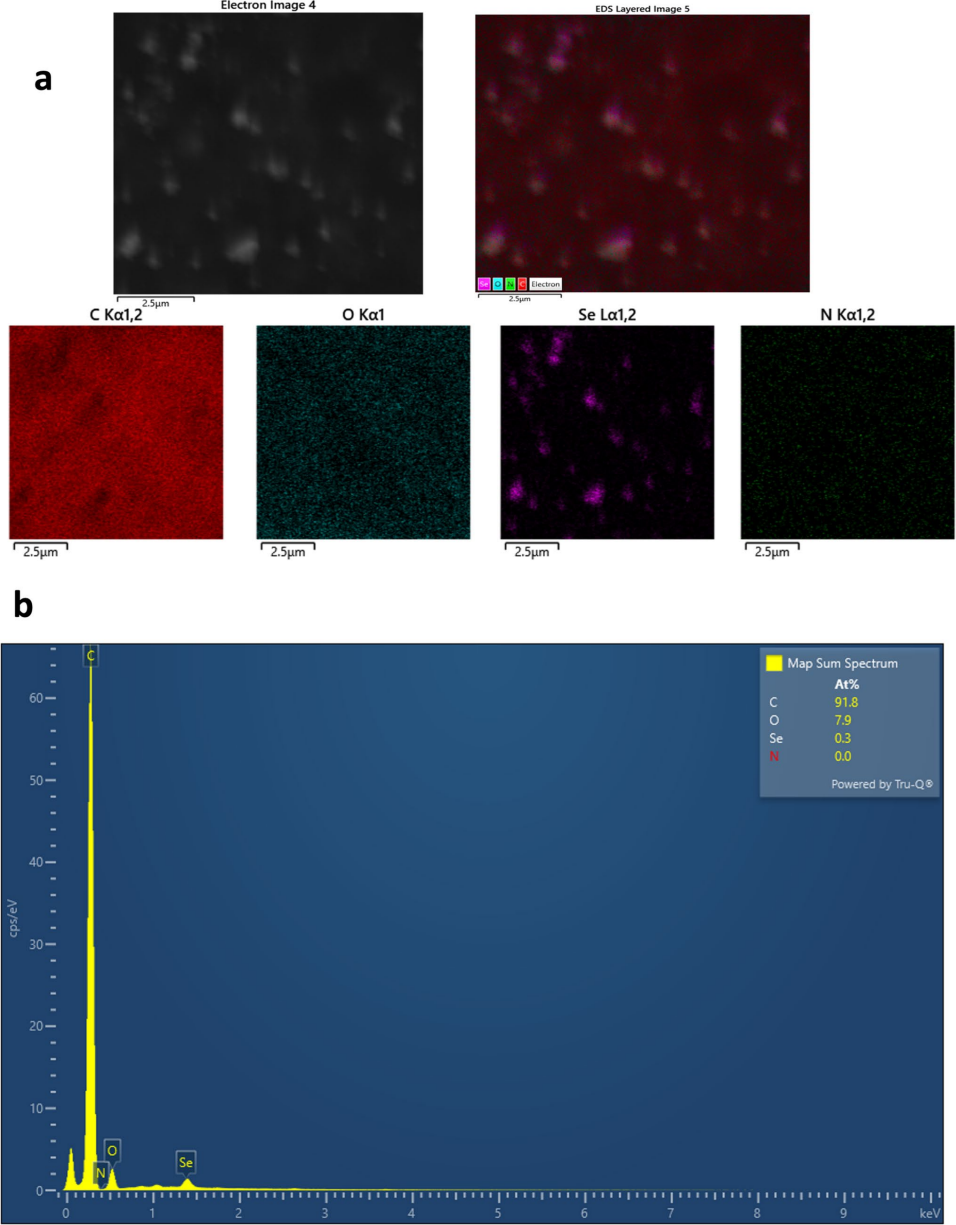
**a** SEM mapping and elemental analysis of Alg@FA-SeNPs and **b** EDX spectrum for Alg@FA-SeNPs Selenium, oxygen, and carbon are uniformly distributed in all formulations, confirming successful synthesis and coating. The lower selenium content in Alg@FA-SeNPs suggests the presence of a protective biopolymer layer, influencing stability and release kinetics

SEM–EDX analysis and elemental mapping confirmed the successful synthesis of Alg@FA-SeNPs. The EDX spectrum revealed the presence of carbon (91.8 At%), oxygen (7.9 At%), and selenium (0.3 At%), consistent with the expected composition of the alginate-coated nanoparticles. The alginate coating was applied to improve the biocompatibility of the nanoparticles and to facilitate a slower, controlled release of ferulic acid. Elemental mapping further demonstrated the uniform distribution of selenium across the nanoparticles, indicating the successful incorporation of selenium into the nanoparticle structure and the integrity of the coating process. The mapping also highlighted the presence of oxygen and carbon, consistent with the alginate matrix surrounding the selenium nanoparticles. These findings collectively confirm that the synthesis and alginate coating of FA-SeNPs were successful, producing nanoparticles with uniform elemental distribution and properties suitable for potential biomedical applications (Fig. 4).

**Fig. 5:**
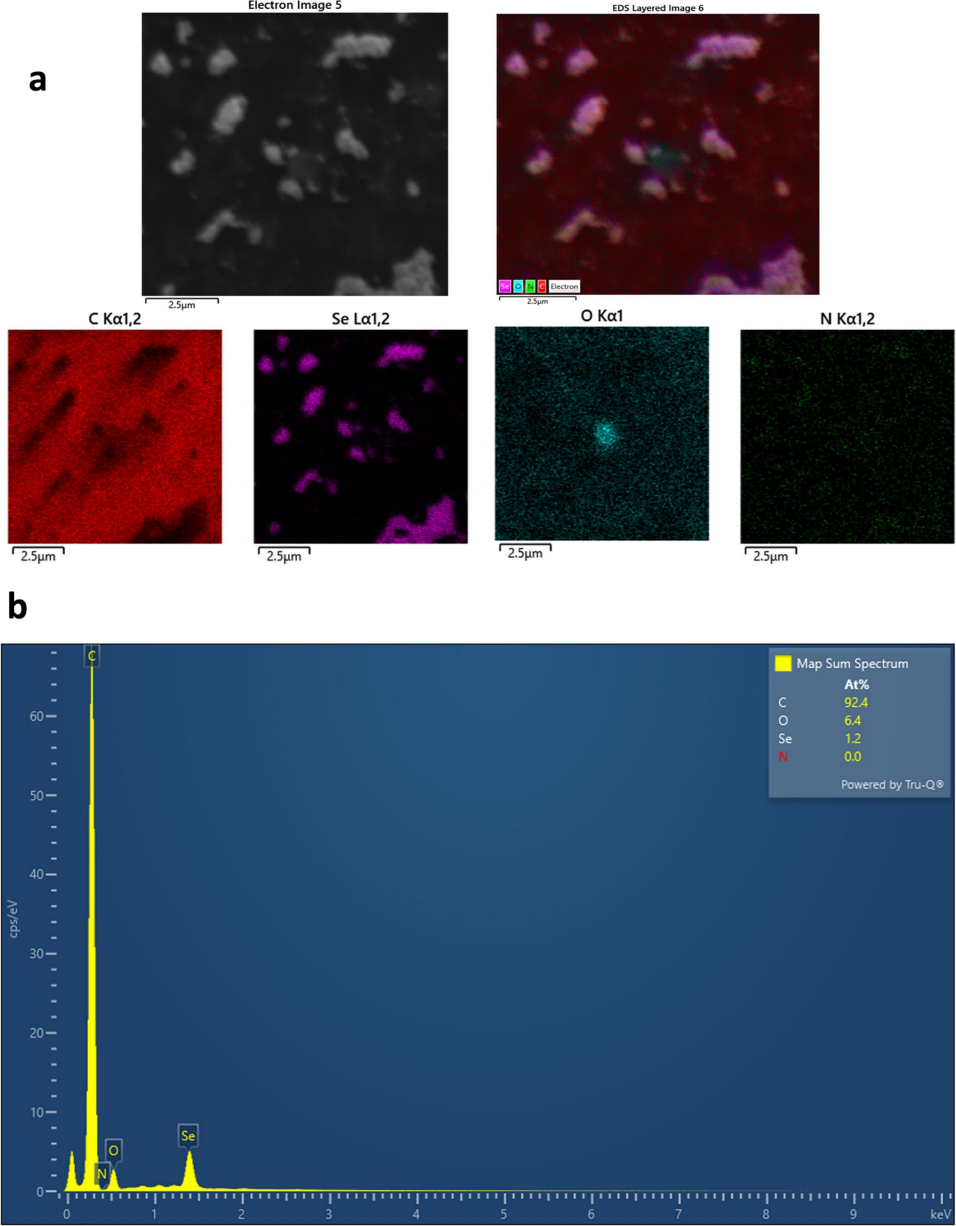
**a** SEM mapping and elemental analysis of CS@FA-SeNPs and **b** EDX spectrum for CS@FA-SeNPs Selenium, oxygen, and carbon are uniformly distributed in all formulations, confirming successful synthesis and coating. The lower selenium level in CS@FA-SeNPs contributes to the formation of biopolymer shell, which influence both nanoparticle stability and release profile

SEM–EDX mapping analysis confirmed the successful synthesis and elemental composition of the CS@FA-SeNPs. The mapping images distinctly show the spatial distribution of key elements, including carbon (C), oxygen (O), and selenium (Se). The dominant presence of carbon (92.4 atomic %) suggested a robust chitosan and ferulic acid coating enveloping the selenium core. Oxygen, constituting 6.4 atomic %, further supported the presence of functional groups contributed by the ferulic acid and chitosan. Selenium was observed at 1.2 atomic %, confirming its incorporation as the nanoparticle core. Notably, nitrogen (N) was not detected (0.0 atomic %). This could be attributed to the masking effect of the graphene layer applied as a surface coating, which may have inhibited the detection of nitrogen signals during the EDX analysis. These results aligned with the structural integrity of the designed nanocarrier system and verified the effective coating of the selenium nanoparticles with chitosan, ferulic acid, and graphene, ensuring the stability and biocompatibility of the nanostructures (Fig. 5).

**Fig. 6:**
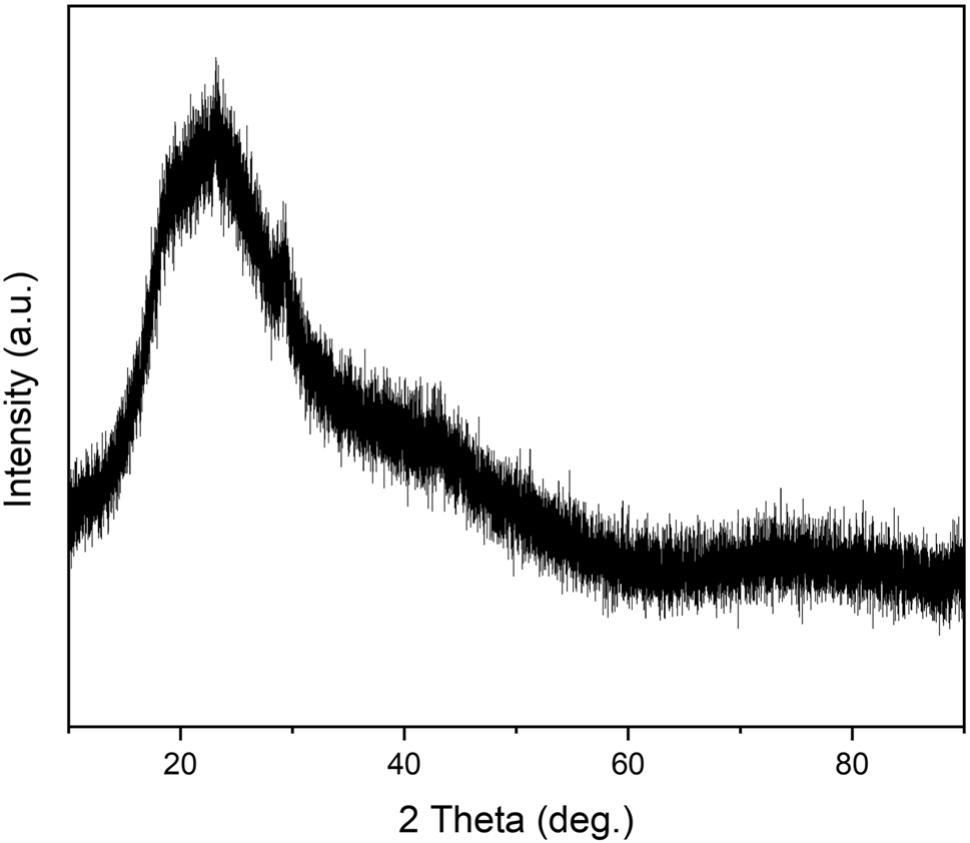
X-ray diffraction (XRD) pattern of the synthesized selenium nanoparticles. The broad halo indicates a predominantly amorphous structure, while low-intensity diffraction peaks at 2*θ* values of 24.03°, 30.3°, 44.2°, 52.2°, and 54.5° suggest the presence of minor crystalline selenium phases

Figure 6 shows the XRD pattern of the synthesized selenium nanoparticles. Upon examination, it is evident that the amorphous structure is dominant; however, low-intensity crystalline peaks can also be observed. The diffraction peaks at 2*θ* values of 24.03°, 30.3°, 44.2°, 52.2°, and 54.5° confirm the presence of crystalline selenium phases. Similar results were observed in the study by Aftab et al. [33], which also utilized selenium nanoparticles. In that study, weak peaks corresponding to elemental selenium were detected alongside a predominantly amorphous structure.

### Drug release of nanoparticles

The drug release profile of Alg@FA-SeNPs over four days showed an initial burst release followed by a sustained release phase in Fig. S2. The initial rapid release was due to surface-bound ferulic acid, while the slower phase resulted from its gradual diffusion through the alginate coating. This controlled release behavior highlighted alginate’s role in maintaining prolonged drug availability, making it suitable for therapeutic applications. CS@FA-SeNPs exhibited a similar two-phase release pattern, with an initial burst followed by sustained release in Fig. S3. The chitosan coating slowed drug release by providing a protective matrix, enhancing stability, and prolonging the release. This sustained behavior supported its potential for cancer therapy by ensuring consistent and localized delivery of the active compound.

### Encapsulation efficiency

The encapsulation efficiency (EE) of FA-SeNP was found to be 64.9%, indicating moderate drug entrapment during the initial formulation. To enhance the retention of the encapsulated drug, the nanoparticles were coated with biopolymers chitosan and alginate. Post-coating analysis revealed that the biopolymer layers preserved almost all of the previously encapsulated drug, with 99.1% and 98.9% of the initial encapsulated ferulic acid retained in the CS@FA-SeNP and Alg@FA-SeNP, respectively. These findings suggest that while the coatings did not further increase the initial encapsulation efficiency, they played a crucial role in maintaining the drug load by preventing leakage and diffusion during processing or storage. This highlights the stabilizing effect of the biopolymer coatings and supports their use in nanoparticle-based drug delivery systems requiring prolonged stability and controlled release.

### MTT assay results: Alg@FA-SeNPs and CS@FA-SeNPs reduce cell proliferation in MDA-MB-231 cells

**Fig. 7:**
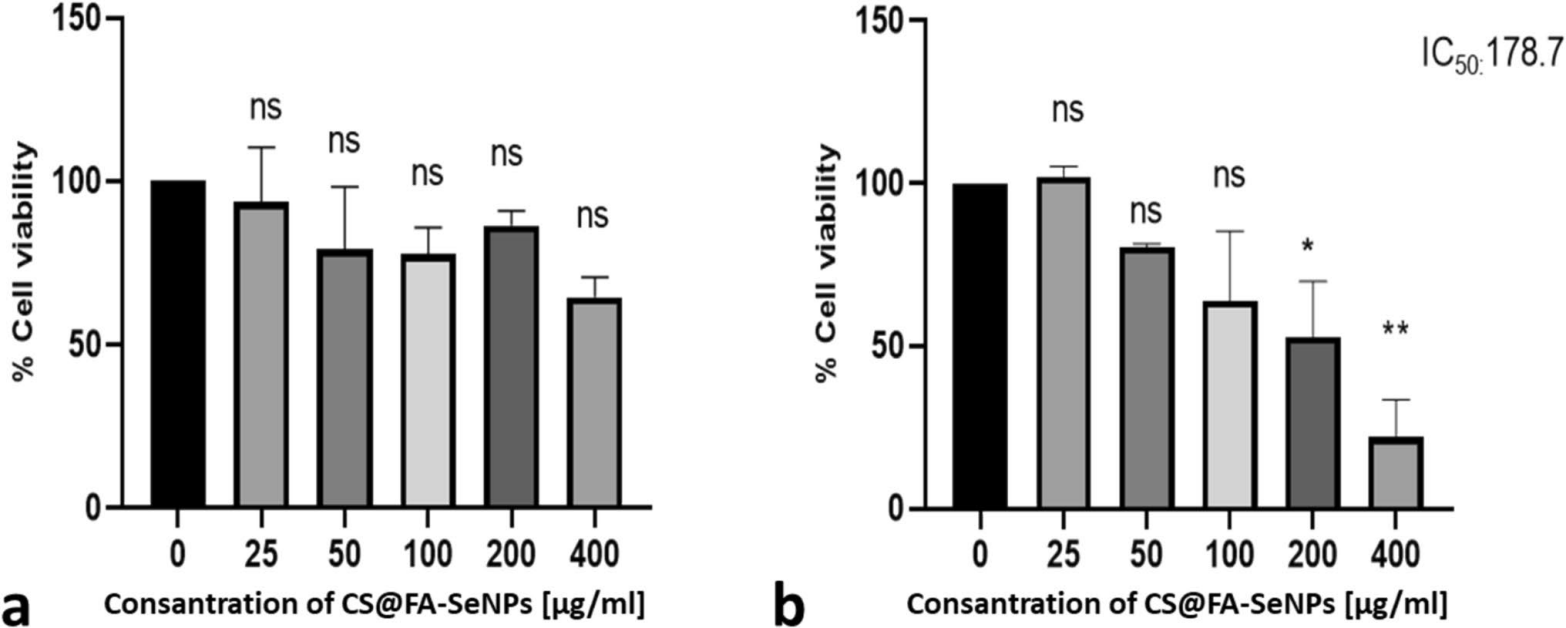
**a** In vitro antiproliferative effect of CS@FA-SeNPs on MDA-MB-231 cells at 24 h. After 24 h, cell viability did not fall below 50% in the dose range studied. **b** At 48 h, cell viability decreased with increasing dose. IC_50_ value was found as 178.7 µg/mL in MDA-MB-231 breast cancer cells treated with CS@FA-SeNPs at 48th hour. The results are reported as cell viability percentage (%) normalized to untreated MDA-MB-231 breast cancer cells. Comparisons between means were performed using one-way ANOVA and Dunnett’s test was utilized for post hoc analysis (*ns* *p* > 0.05, *p ≤ 0.05, **p ≤ 0.01, ***p ≤ 0.001, and ****p ≤ 0.0001)

The present study sought to demonstrate the cytotoxic effect of Alg@FA-SeNPs and CS@FA-SeNPs on MDA-MB-231 triple-negative breast cancer cells. To this end, the colorimetric-based MTT technique was employed to measure the effect of varying doses and times. The examination of cell viability at concentrations ranging from 25 to 400 µg/mL revealed a dose-dependent decrease in MDA-MB-231 breast cancer cells under in vitro conditions, concomitant with an increase in nanoparticle concentration. According to MTT results, cell viability in MDA-MB-231 breast cancer cells treated with CS@FA-SeNPs did not fall below 50% in the dose range studied at 24 h. At 48 h, it was observed that cell viability also decreased depending on the increasing dose. Utilizing GraphPad Prism 8 software, the IC50 value for CS@FA-SeNPs in MDA-MB-231 breast cancer cells at the 48-h timepoint was determined to be 178 µg/mL, as illustrated in Fig. 7a and b.

**Fig. 8:**
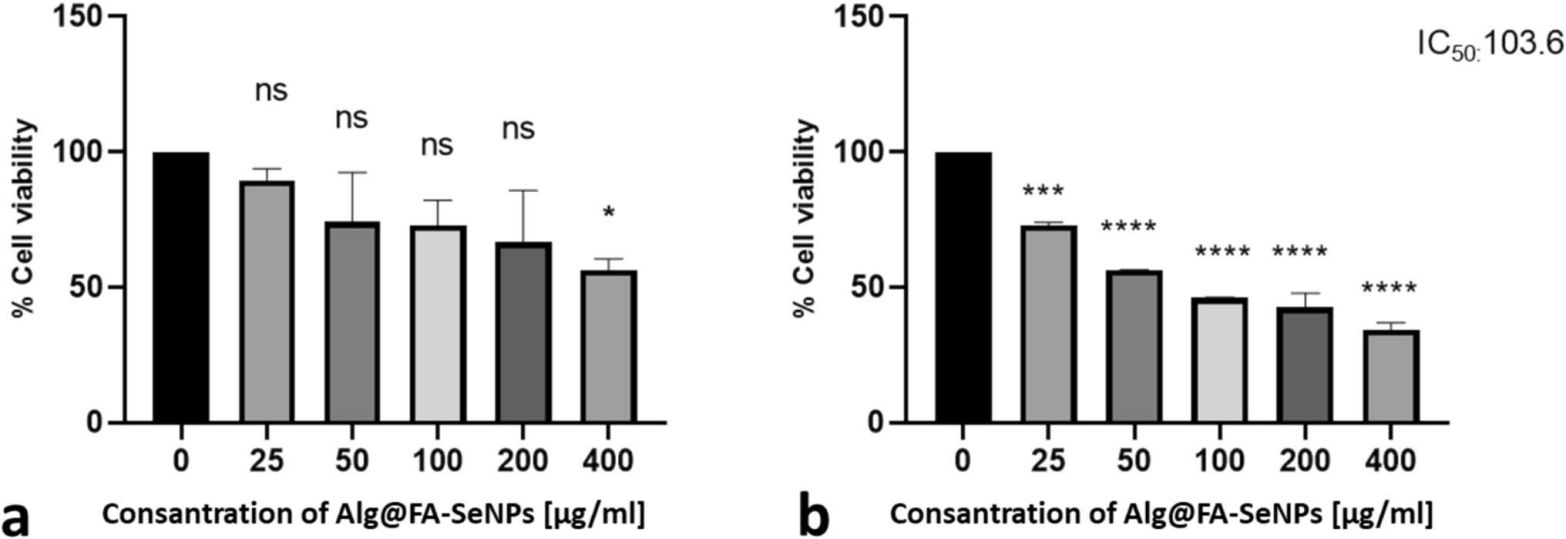
**a** In vitro antiproliferative effect of Alg@FA-SeNPs on MDA-MB-231 cells at 24 h. After 24 h, cell viability did not fall below 50% in the dose range studied. **b** At 48 h, cell viability decreased with increasing dose. IC_50_ value was found as 103.6 µg/mL in MDA-MB-231 breast cancer cells treated with Alg@FA-SeNPs 48th hour. The results are reported as cell viability percentage (%) normalized to untreated MDA-MB-231 breast cancer cells. Comparisons between means were performed using one-way ANOVA and Dunnett’s test was utilized for post hoc analysis (*ns* *p* > 0.05, *p ≤ 0.05, **p ≤ 0.01, ***p ≤ 0.001, and ****p ≤ 0.0001)

Similar results were obtained for Alg@FA-SeNPs. According to MTT results, cell proliferation at the end of 24 h decreased in all dose groups compared to the control, but the cell viability rate could not fall below 50%. However, cell proliferation at 48 h was significantly reduced and the IC50 dose was determined to be 103.6 µg/mL (Fig. 8a, b). Both chitosan and alginate-coated ferulic acid selenium nanoparticles reduced cell proliferation in MDA-MB-231 breast cancer cells at 48 h and showed similar antiproliferative effects. While the Ic50 value of CS@FA-SeNPs was determined to be 178 µg/mL, this value was slightly lower and 103.6 µg/mL for Alg@FA-SeNPs.

### Real-time PCR results: Alg@FA-SeNPs and CS@FA-SeNPs regulate mRNA expressions of apoptosis-related genes in MDA-MB-231 cells

Expression changes at the mRNA level of apoptosis-related genes *caspase-3, caspase-8, caspase-9, Bax, Bcl-2* genes and genotoxicity-related *H2AX* and oxidative stress-related *NRF2* genes were detected by real-time PCR method (Table 2). In MDA-MB-231 breast cancer cells treated with CS@FA-SeNPs, *caspase-8, caspase-9*, and *H2AX* genes were increased by 1.72-fold, 1.26-fold and 1.52-fold, respectively, compared with the control, and these increases were not found to be statistically significant (*p* > 0.05). Similarly, the expression changes of caspase-3 and Bax genes did not change after treatment with CS@FA-SeNPs, and in this case, it was not found to be statistically significant. The expression of *Bcl-2*, one of the important markers of apoptosis and one of the anti-apoptotic genes, was significantly decreased and this change was found to be statistically significant (*p* = 0.004408). In the CS@FA-SeNPs nanoparticle dose group, the fold change value of Bcl-2 gene was determined to be 0.38. Similarly, *NRF2* gene expression was also decreased in the CS@FA-SeNPs nanoparticle dose group, and the fold change value was determined to be 0.33, but this change was not found to be statistically significant. (Table 2).

**Table 2:** mRNA expression fold changes and *p* values of apoptosis and oxidative stress-related genes in MDA-MB-231 breast cancer cells after CS@FA-SeNPs and Alg@FA-SeNPs treatment (*p < 0.05)

Gene names	*CS@FA-SeNPs*		*Alg@FA-SeNPs*	
	Fold change	*p* value	Fold change	*p* value
*Caspase-3*	1.11	0.724409	1.64	0.098039
*Caspase-8*	1.72	0.230873	1.60	0.147893
*Caspase-9*	1.26	0.959124	3.19	0.129905
*Bax*	1.05	0.877938	0.57	0.219056
*Bcl-2*	0.38	0.004408*	1.22	0.225373
*H2aX*	1.52	0.247360	4.45	0.014850*
*NRF2*	0.33	0.149460	1.91	0.577420

In the Alg@FA-SeNPs nanoparticle dose group, only *Bax* expression decreased, and other gene expressions increased compared to the control. Although the 1.64-, 1.60-, and 3.19-fold increases in *caspase-3, caspase-8, and caspase-9* genes, respectively, were remarkable, they were not statistically significant (*p* > 0.05). A 1.91-fold increase in *NRF2* gene was observed in MDA-MB-231 cells treated with Alg@FA-SeNPs, and this change was not statistically significant. *H2AX* expression, one of the important genotoxicity markers, showed a significant 4.45-fold increase after exposure to Alg@FA-SeNPs and this change was statistically significant (*p* = 0.014850) (Table 2).

### TAS-TOS and OSI results: Alg@FA-SeNPs and CS@FA-SeNPs effect total oxidant, total antioxidant, and oxidative stress parameters in MDA-MB-231 cells

**Fig. 9:**
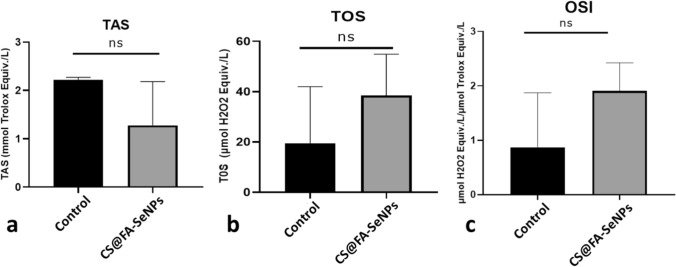
**a** TAS, **b** TOS, and **c** OSI levels in MDA-MB-231 breast cancer cells after CS@FA-SeNPs treatment (*p* > 0.05, *ns* non-significant)

The effects of indigenously synthesized Alg@FA-SeNPs and CS@FA-SeNPs on total oxidant, total antioxidant, and oxidative stress parameters were determined using TAS-TOS method on MDA-MB-231 triple-negative breast cancer cells under in vitro conditions. When the results obtained were examined, total oxidant capacity in breast cancer cells treated with CS@FA-SeNPs decreased by half compared to the control. Total oxidant effect increased twice compared to the control. In all these cases, the oxidative stress index increased nearly twofold with the treatment with CS@FA-SeNPs. However, the data obtained were not statistically significant (Fig. 9a–c).

**Fig. 10:**
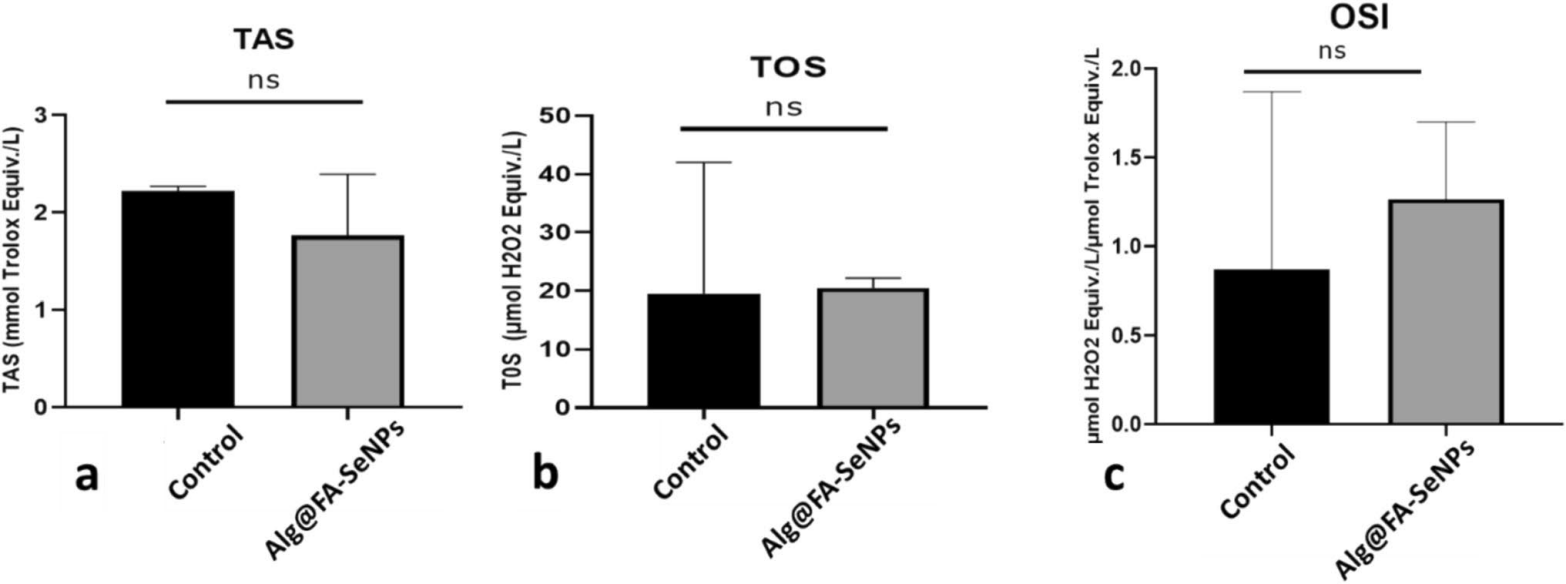
**a** TAS, **b** TOS, and **c** OSI levels in MDA-MB-231 breast cancer cells after Alg@FA-SeNPs nanoparticle treatment (*p* > 0.05, *ns* non-significant)

A similar situation was observed in breast cancer cells treated with Alg@FA-SeNPs. Total oxidant capacity decreased by half compared to the control. However, total oxidant effect did not show any change in breast cancer cells treated with Alg@FA-SeNPs compared to control. In all these cases, a partial increase in oxidative stress index was observed with the treatment with Alg@FA-SeNPs. However, the changes obtained were not statistically significant (Fig. 10a–c).

As a result of TAS and TOS experiments, it was determined that the oxidative stress index increased in the dose group cells compared to the control as a result of the treatment of the nanoparticles synthesized originally.

## Discussion

This study demonstrates the potential of selenium nanoparticles (SeNPs) as a nanoplatform for the targeted delivery of ferulic acid to breast cancer cells. Our aim was to understand how different surface coatings influence cellular responses. The unique characteristics of Alg@FA-SeNPs and CS@FA-SeNPs highlight the significance of coating materials in affecting the stability, cellular absorption, and therapeutic effectiveness of nanoparticles. Alginate, recognized for its gelling and biocompatibility characteristics, promoted increased anticancer efficacy at reduced concentrations, presumably owing to its improved controlled release profile. Chitosan, possessing mucoadhesive and antibacterial characteristics, yielded a strong coating but necessitated elevated doses for comparable effects, possibly because of variations in release kinetics. The results revealed that CS@FA-SeNPs and Alg@FA-SeNPs, despite having identical core compositions, elicited distinct cellular effects, indicating that the surface chemistry of nanoparticles plays a critical role in determining their intracellular behavior and therapeutic potential.

The drug release profiles of Alg@FA-SeNPs and CS@FA-SeNPs at 27 °C (Figs. S4, S5) revealed distinct release patterns influenced by the coating materials. Alg@FA-SeNPs exhibited a biphasic release with an initial burst in the first 2 h, followed by a sustained release phase, and a secondary increase between 48 and 96 h. Release was slightly higher in acetate buffer (pH 5.0), likely due to alginate’s moderate pH sensitivity, which promotes swelling and diffusion under acidic conditions.

In contrast, CS@FA-SeNPs showed a rapid burst release in the first hour, especially in PBS, followed by a plateau. Although chitosan is more soluble under acidic conditions, the overall release remained lower in acetate buffer, possibly due to tighter polymer packing or stronger drug interactions limiting diffusion.

The drug release profile of CS@FA-SeNPs at 37 °C (Fig. S3) showed an initial burst in both acetate buffer and PBS, followed by a slower, sustained release. The initial rapid release likely stems from surface-associated drug. Subsequently, release slowed, suggesting diffusion-controlled release. Interestingly, the cumulative drug release appeared slightly higher in PBS compared to the acetate buffer over the longer duration. This observation is somewhat counterintuitive, as chitosan’s increased solubility and swelling at lower pH values typically promote faster drug release. The potentially lower release in the acetate buffer could be attributed to other factors, such as stronger electrostatic interactions between the protonated chitosan and the drug, or a different equilibrium of drug partitioning within the nanoparticle matrix at the lower pH. The significant initial burst warrants optimization for controlled delivery, such as modifying drug loading or the coating. The subtle differences in release profiles under different pH conditions underscore the importance of considering the target physiological environment. Further studies should investigate the interplay of pH and drug–polymer interactions on the release mechanism and in vivo performance. The drug release profile of Alg@FA-SeNPs (Fig. S2) demonstrated a biphasic release pattern in both physiological (PBS, pH 7.4) and acidic (acetate buffer, pH 5.0) conditions at 37 °C, over 96 h. An initial burst release was observed within the first 2 h, followed by a relatively sustained release phase extending up to 72 h, and culminating in a secondary increase in cumulative drug release between 72 and 96 h. This early release is likely attributed to the diffusion of surface-associated or loosely bound ferulic acid molecules. The slightly higher release rate in the acetate buffer, particularly during the burst and final stages, may be due to the protonation of alginate’s carboxyl groups under acidic pH, which enhances polymer swelling or partial degradation. These results suggest that the alginate matrix exhibits moderate pH sensitivity, favoring increased drug diffusion under acidic conditions typical of tumor microenvironments.

Importantly, the sustained release observed over 96 h provides a plausible mechanistic basis for the heightened cytotoxicity seen in the MDA-MB-231 cell line. While neither formulation significantly altered oxidative stress parameters, the controlled and prolonged release of ferulic acid and selenium may enable continuous intracellular exposure, leading to cumulative genotoxic stress. This aligns with the observed upregulation of *H2AX,* a key marker of DNA damage, in cells treated with Alg@SeNP-FA. The late-phase burst after 72 h may further amplify intracellular drug accumulation at later time points, reinforcing DNA damage pathways without requiring ROS-mediated cytotoxicity. Collectively, these findings highlight the critical role of release kinetics in determining therapeutic efficacy. Alginate appears to offer a suitable matrix for pH-responsive, sustained release, thereby enhancing the bioavailability and nuclear effects of encapsulated agents like ferulic acid in triple-negative breast cancer cells.

The Alg@FA-SeNPs demonstrated significantly higher cytotoxicity compared to CS@FA-SeNPs. This increased cytotoxicity was accompanied by a marked upregulation of *H2AX* gene expression, a well-established marker of DNA double-strand breaks and replication stress [34]. Notably, these effects were observed without significant alterations in oxidative stress markers, including total oxidant status (TOS), total antioxidant status (TAS), or the oxidative stress index (OSI). This finding suggests that the genotoxic stress induced by Alg@FA-SeNPs operates through a mechanism that is independent of global reactive oxygen species (ROS) generation. One plausible explanation is that the alginate coating facilitates a more sustained release of ferulic acid and selenium, allowing for prolonged intracellular exposure that may interfere with DNA replication or chromatin stability. Alternatively, the alginate matrix might influence nanoparticle trafficking, enabling greater nuclear accumulation or interaction with nuclear proteins. Given that H2AX phosphorylation is a sensitive response to DNA damage [35], the upregulation observed here likely reflects nanoparticle-induced replication fork stalling or chromatin disruption rather than oxidative insult. This suggests a ROS-independent genotoxic pathway mediated by nanoparticle-DNA or protein interactions.

In contrast, CS@FA-SeNPs exhibited lower cytotoxicity but selectively downregulated *Bcl-2*, an anti-apoptotic gene involved in mitochondrial integrity. The suppression of *Bcl-2* indicates the initiation of intrinsic apoptotic signaling. The lack of corresponding upregulation of oxidative stress markers suggests that the CS@FA-SeNPs may prime cells for apoptosis without fully activating the cell death machinery. This response could be due to the cationic nature of chitosan, which enhances cellular uptake and preferentially localizes nanoparticles to endosomal or mitochondrial compartments, thereby affecting mitochondrial membrane potential and downstream apoptotic signaling.

Interestingly, despite the known antioxidant and pro-oxidant duality of selenium and ferulic acid, neither formulation significantly altered TAS, TOS, or OSI levels in MDA-MB-231 cells. This underscores the importance of evaluating localized or pathway-specific cellular responses, as global oxidative stress assays may not detect subtle but biologically meaningful intracellular events. The lack of oxidative stress supports a growing body of evidence that nanoparticle-mediated cytotoxicity may occur through alternative mechanisms, including direct genotoxicity, epigenetic modulation, or interference with signaling cascades.

Collectively, these findings reveal that surface coating materials can fundamentally alter the intracellular fate and biological activity of therapeutic nanoparticles, even when the core composition remains constant. Alginate coatings appear to direct the nanoparticle payload toward nuclear and genotoxic effects, while chitosan coatings modulate mitochondrial apoptotic signaling. These insights are particularly valuable for designing targeted nanomedicine strategies, as different coatings may be employed selectively depending on whether genotoxic stress or apoptotic sensitization is the desired therapeutic outcome. Moreover, chitosan-coated formulations that modulate apoptosis-related genes without causing significant toxicity may hold promise as adjuvants in combination therapies, where they could enhance the efficacy of conventional chemotherapeutic agents.

Future studies should further investigate the intracellular trafficking and release kinetics of these nanoparticle systems, as well as expand gene expression profiling to include broader apoptosis and DNA damage pathways. Understanding the subcellular distribution and temporal behavior of these formulations will be essential for optimizing their design and maximizing their clinical utility.

We evaluated the encapsulation efficiency (EE) and retention of ferulic acid (FA) within selenium nanoparticles (SeNPs), both uncoated and coated with biopolymers. The uncoated FA-SeNPs exhibited an EE of 64.9%, aligning with previous reports where FA was incorporated into nanoparticulate systems. For instance, they demonstrated the successful synthesis of FA-SeNPs, highlighting their potential in enhancing antitumor activities. While their study primarily focused on the biological effects, the incorporation of FA into SeNPs is consistent with our approach.

Upon coating the FA-SeNPs with chitosan and alginate, we observed a significant improvement in drug retention, with 99.1% and 98.9% of the initially encapsulated FA retained, respectively. This enhancement can be attributed to the barrier properties of the biopolymer coatings, which likely minimized drug leakage.

The zeta potential of the Alg@FA-SeNPs (Fig. S6) was measured to assess their colloidal stability. The recorded values were − 2.51 mV (SD: 5.21 mV) for sample 1, − 2.07 mV (SD: 5.14 mV) for sample 2, and − 2.04 mV (SD: 4.22 mV) for sample 3. These near-neutral zeta potentials indicate a limited surface charge, which may be attributed to the presence of calcium ions (Ca^2^⁺) used during the coating or stabilization process. Ca^2^⁺ ions can bind to the negatively charged carboxylate groups on the alginate backbone, neutralizing the surface charge and thereby reducing the overall zeta potential. This suggests that the electrostatic stability of the nanoparticles is influenced not only by the alginate coating but also by the ionic interactions involving divalent cations.

The surface charge of the CS@FA-SeNPs (Fig. S7) was determined by zeta potential measurements. The recorded values from three independent runs were + 24.4 mV, + 24.5 mV, and + 23.9 mV, respectively. These consistently positive values confirm the effective coating of selenium nanoparticles with chitosan, which is known to impart a positive surface charge due to its protonated amino groups. Average zeta potential exceeding + 24.3 mV is generally indicative of good colloidal stability, as the electrostatic repulsion between particles reduces aggregation tendencies. Moreover, the strong positive surface charge may promote enhanced interaction with negatively charged cellular membranes, potentially improving cellular internalization.

Aggregation significantly impacts the drug release and stability of nanoparticles, potentially reducing therapeutic efficacy. When nanoparticles aggregate, their surface area decreases, slowing or disrupting drug release and promoting premature sedimentation or immune recognition. This process can lead to reduced bioavailability and rapid clearance. To prevent aggregation, strategies like surface modification, concentration optimization, and controlled storage are essential. Maintaining colloidal stability is crucial for consistent drug delivery and maximizing therapeutic potential.

Ria and their team suggest that dietary polyphenol ferulic acid promoted epirubicin-induced ER stress-induced MDA-MB-231 breast cell mortality [36]. The combined index of 10 μM ferulic acid and epirubicin was below 0.9, indicating increased MDA-MB-231 cell sensitivity to epirubicin. A flow cytometry study demonstrated that ferulic acid (10 μM) boosted apoptosis in MDA-MB-231 cells treated with epirubicin (1 μM). Increased Bax, decreased Bcl-2, and caspase-3 caused apoptosis in western blots. Ferulic acid (10 μM) and epirubicin (1 μM) elevated ER stress-related protein expression, including PDI, IRE1α, and PEPK, while ER stress inhibitors had the reverse effect Ferulic acid may help breast cancer. Ferulic acid-loaded polymeric and lipidic nanocapsules were produced and analyzed in another study [37]. Lipidic nanocapsules outperformed polymeric nanocapsules cellularly, and an in vivo colorectal cancer model showed their therapeutic potential. The study highlights the importance of nutraceuticals like ferulic acid in cancer treatment and the promise of lipidic nanocapsules as a delivery vehicle to enhance its anticancer activities. This study [38] produced stable gold nanoparticles from polyphenol ferulic acid. The process involved the reduction of hydrogen tetrachloroaurate (III) hydrate at room temperature. Spherical Fa-stabilized gold nanoparticles. A431 human skin cancer cells and HaCaT normal keratinocytes were tested for Fa-AuNPs. A431 cells were cytotoxically harmed by Fa-AuNPs depending on dose and duration. CAM assessed Fa-AuNPs’ angiogenetic efficacy. Apoptosis is suggested by sub G1 cells. High ROS and caspase-3 reduced mitochondrial membrane potential. Mitochondria-based pathways killed A431 cells, suggesting fa-AuNPs may treat skin cancer.

Despite its antioxidant and anticancer properties, ferulic acid (FA) has low bioavailability due to its low water solubility [39]. FA-loaded polymeric mixed micelles will overcome its poor solubility and test colon cancer antitumor with miRNA-221/TP53INP1 axis-mediated autophagy. Polymeric mixed micelles were improved using a D-optimal design with three FA and TPGS mixed micelles formula (O2) were investigated for anticancer activity using MTT and flow cytometry to determine how total Pluronics mixture (mg), Pluronic P123 percentage (%w/w), and drug amount (mg) affected entrapment efficiency (EE%) and particle O2 contained 13.86 nm particles, 99.89% EE%, and − 6.02 mv zeta potential. O2-released FA was faster than free FA in in vitro drug release tests. IC50 values for FA from O2 and free FA against Caco-2 were significant (17.1 and 191 µg/mL, respectively) in many cell lines. Flow cytometry showed that FA suppressed Caco-2 G2/M cell cycle. Solubility may improve FA’s colorectal cancer anticancer activities in FA-loaded TPGS mixed micelles. TPGS mixed micelles with FA may cure colorectal cancer by targeting miRNA-221/TP53INP1 axis-mediated autophagy. Pruthi and colleagues [40] encapsulated FA in CS-TPP NPs to boost its therapeutic potential. They investigated several FA concentrations to get the best-sized FA-loaded CS-TPP nanoparticles (FA/NPs) by ionic gelation. The cytotoxicity of 40 µM FA/CS-TPP NPs against ME-180 cervical cancer cell lines was higher than that of native FA. ME-180 cells showed apoptotic morphological alterations such as cytoplasmic remains and damaged wrinkled cells using scanning electron and fluorescence microscopy. Data showed that chitosan-enveloped FA nanoparticles could treat cancer cell growth. Li and groups [41] found that FA-SeNPs suppressed HepG-2 cell growth with an IC50 of 11.57 ± 3.6 μg/mL, while Se NPs had an IC50 of > 100 μg/ml. FA does not exhibit anticancer effects at 100 μg/mL doses. HepG-2 cell death was assessed using fluorescence morphology and Annexin V-FITC/PI staining to investigate FA-SeNPs’ anticancer mechanism. ROS, MMP, and caspase-3 and -9 activities were measured. FA-SeNPs activated caspase-3/9 to kill mitochondrial HepG-2 cells by disrupting MMPs and creating ROS. Further investigation of calf thymus DNA suggests FA-SeNPs’ DNA-binding capabilities may fight cancer. Our study demonstrated the superior efficacy of selenium nanoparticles coated with biopolymers like alginate and chitosan, particularly in breast cancer. Notably, the alginate and chitosan coating facilitated more controlled drug release, potentially enhancing the nanoparticles’ residence time within the tumor and efficacy.

IC50 values have been reported for various chemotherapeutics in the MDA-MB-231 cell line. For example, doxorubicin (DOX), commonly used in breast cancer treatment, has shown variable IC50 values in experimental settings. In MDA-MB-231 cells, doxorubicin exhibits an IC50 of approximately 0.83869 µg/mL [42]. Cisplatin, another commonly used chemotherapeutic, has also been evaluated for its cytotoxic effects on MDA-MB-231 cells. One study showed an IC50 value of approximately 12 µM for cisplatin when analyzed through cell viability assays [43]. While the IC50 value of CS@FA-SeNPs was determined as 178 µg/mL, this value was slightly lower and 103.6 µg/mL for Alg@FA-SeNPs in MDA-MB-231 cells. Although the nanoparticles that we synthesized in our study show effects at higher dose concentrations compared to existing chemotherapeutic agents, this has the potential to increase their efficacy when applied in combination with existing chemotherapeutics. The variability in these values underlines the potential for tailored therapeutic approaches depending on cellular characteristics and the underlying resistance mechanisms operating in individual tumors.

The limitations of our study, including the lack of in vivo data and comprehensive mechanistic insights, highlight the need for further investigations. Another limitation of this study is the lack of a healthy non-cancer cell line. Limitations of this study also include the lack of protein level analysis (such as western blot) of other oxidative stress markers, the lack of genotoxicity studies, and flow cytometric analysis confirmation. Future work should explore the pharmacokinetics and biodistribution of these nanoparticles in animal models, along with their synergistic potential with other anticancer agents. Overall, alginate-chitosan FA-SeNPs may treat triple-negative breast cancer. Dose- and time-dependent antiproliferative effects of lower Alg@FA-SeNP concentrations were greater than CS@FA-SeNPs. Apoptosis markers and oxidative stress responses were altered by nanoparticles in gene expression studies, suggesting they could kill cells and decrease cancer progression. Nanoparticles may alter cancer cell redox equilibrium, even while oxidative stress was not considerable. These results imply biopolymer-coated SeNPs increase cancer treatment bioavailability, stability, and targeting.

Nevertheless, nanoparticle accumulation in non-cancerous tissues is a critical concern in nanomedicine, as it can lead to unintended toxicity and adverse effects. The biodistribution of nanoparticles depends on factors such as size, surface charge, coating materials, and administration route. While nanoparticle modifications, such as surface functionalization with biopolymers or targeting ligands, can enhance specificity to cancer cells, off-target accumulation in organs like the liver, spleen, and kidneys remains a challenge. To improve safety, strategies such as using biodegradable nanoparticles, optimizing dosing regimens, and incorporating stimuli-responsive release mechanisms are being explored. Additionally, thorough in vivo toxicity studies and long-term assessments are essential to ensure the biocompatibility and safe clinical translation of nanoparticle-based therapies.

## Conclusion

This study demonstrated the successful synthesis and characterization of ferulic acid-loaded selenium nanoparticles (FA-SeNPs) coated with alginate (Alg@FA-SeNPs) and chitosan (CS@FA-SeNPs) for targeted therapy against triple-negative breast cancer (TNBC). Both formulations showed dose- and time-dependent antiproliferative effects, with Alg@FA-SeNPs exhibiting greater efficacy (IC50: 103.6 µg/mL) than CS@FA-SeNPs (IC50: 178 µg/mL). Gene expression analysis revealed that Alg@FA-SeNPs significantly upregulated H2AX (4.45-fold, *p* = 0.015), indicating DNA damage, while CS@FA-SeNPs markedly downregulated Bcl-2 (0.38-fold, *p* = 0.004), promoting apoptosis. Furthermore, multi-omics analyses—including transcriptomics and proteomics—may offer deeper insights into the molecular mechanisms triggered by these nanocarriers. The biopolymer coatings improved nanoparticle stability, enabled sustained drug release, and provided favorable surface properties, as confirmed by zeta potential and XRD analyses. These findings emphasize the importance of surface chemistry in modulating the biological performance of nanocarrier systems and highlight the clinical potential of FA-SeNPs as targeted nanocarriers, warranting further in vivo studies to support their translation into therapeutic applications for TNBC.

Future studies should assess the in vivo efficacy and safety of these nanoparticles, particularly in TNBC animal models. In addition, optimization of polymer concentration and the use of targeting ligands may enhance tumor-specific delivery and minimize off-target effects.

## Supplementary Information

Below is the link to the electronic supplementary material.

Supplementary file1 (DOCX 2916 KB)

## Data Availability

No datasets were generated or analyzed during the current study.
