# Supplementary material for: Alginate and chitosan-coated ferulic acid-loaded selenium nanoparticles: synthesis, characterization, and anticancer activity against MDA-MB-231 breast cancer cells
Source: Med Oncol. 2025 May 5;42(6):198. doi: 10.1007/s12032-025-02756-8 (PMC12053028; doi:10.1007/s12032-025-02756-8)
Supplement: Supplementary file 1 — Supplementary file1 (DOCX 2916 KB) [file 12032_2025_2756_MOESM1_ESM.docx]

**Supplementary Information**

**Alginate and Chitosan-Coated Ferulic Acid-Loaded Selenium Nanoparticles: Synthesis, Characterization, and Anticancer Activity Against MDA-MB-231 breast cancer cells.**

**The Particle Size Distribution Analysis**


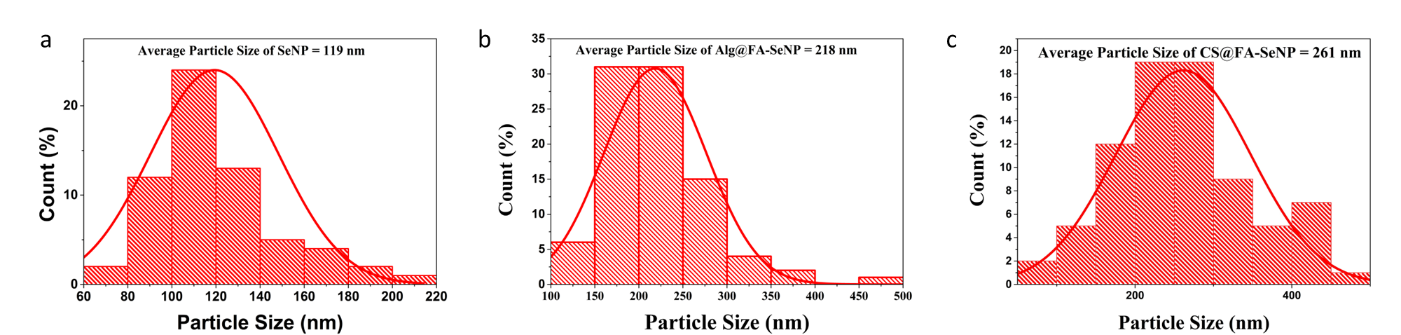


**Fig. S1** Particle size distribution histograms of a SeNPs, b Alg@FA-SeNPs, and c CS@FA-SeNPs. The average particle sizes were determined to be 119 nm, 218 nm, and 261 nm for SeNPs, Alg@FA-SeNPs, and CS@FA-SeNPs, respectively.

**Drug Release Profile Of Alg@FA-SeNPs at 37°C**


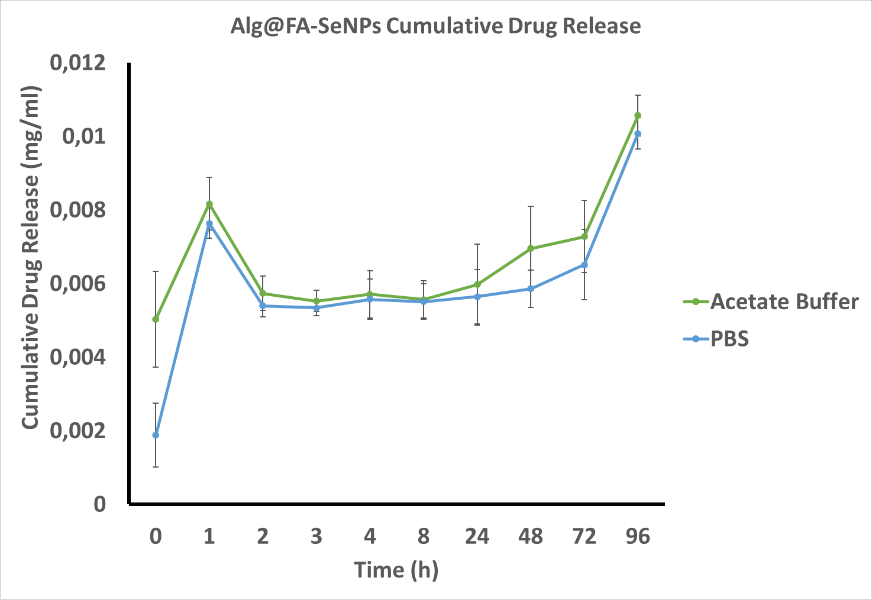


**Fig. S2** Drug release profiles of Alg@FA-SeNPs over four days at 37°C. Drug release experiments were performed in triplicate, each consisting of three technical replicates, and the results are shown as mean ± SEM. Error bars represent the standard error of the mean.

**Drug Release Profile Of CS@FA-SeNPs 37°C**


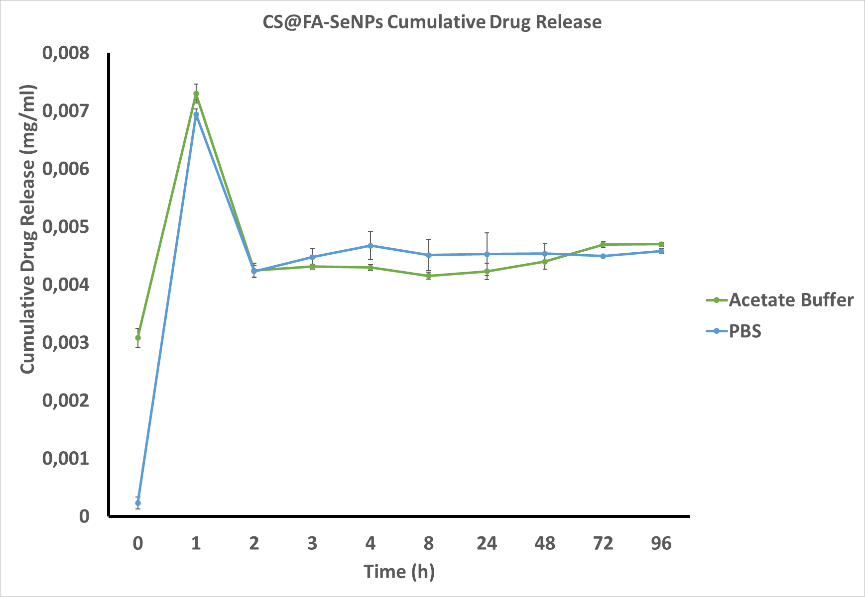


**Fig. S3** Drug release profiles of CS@FA-SeNPs over 4 days at 37°C. Drug release experiments were performed in triplicate, each consisting of three technical replicates, and the results are shown as mean ± SEM. Error bars represent the standard error of the mean.

**Drug Release Profile Of Alg@FA-SeNPs at 27°C**

**
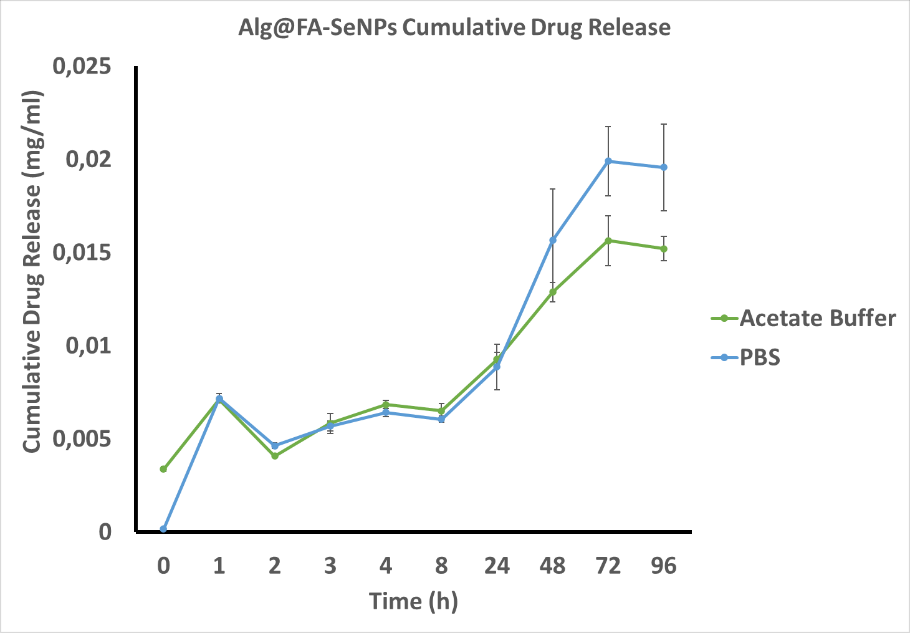
**

**Fig. S4** Drug release profiles of Alg@FA-SeNPs over 4 days at 27°C. Drug release experiments were performed in triplicate, each consisting of three technical replicates, and the results are shown as mean ± SEM. Error bars represent the standard error of the mean.

**Drug Release Profile Of CS@FA-SeNPs at 27°C**


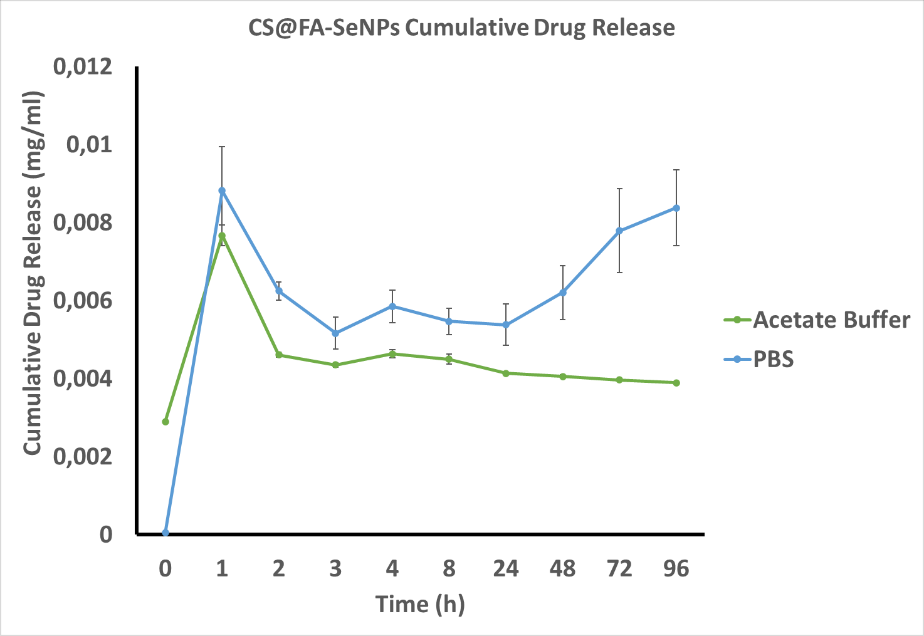


**Fig. S5** Drug release profiles of CS@FA-SeNPs over 4 days at 27 °C. Drug release experiments were performed in triplicate, each consisting of three technical replicates, and the results are shown as mean ± SEM. Error bars represent the standard error of the mean.

**Zeta Potential of Alg@FA-SeNPs**


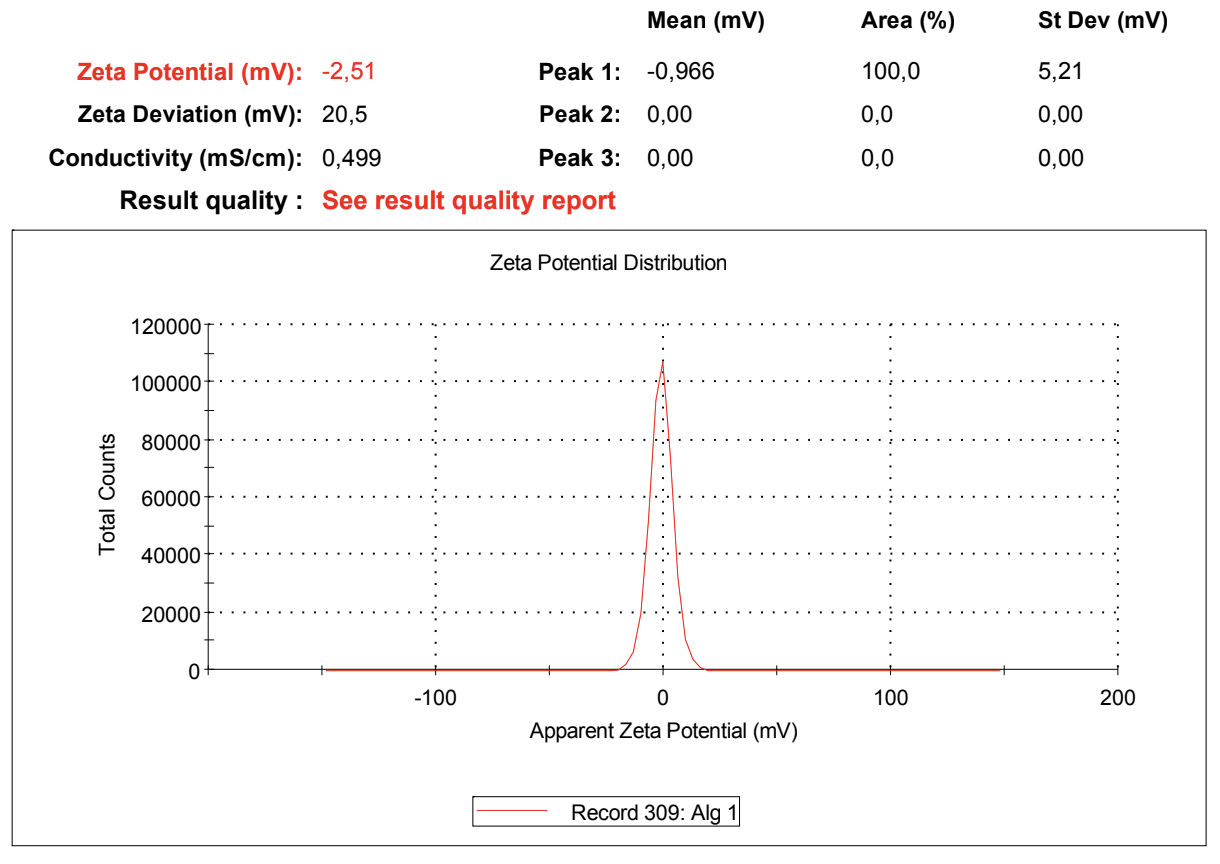

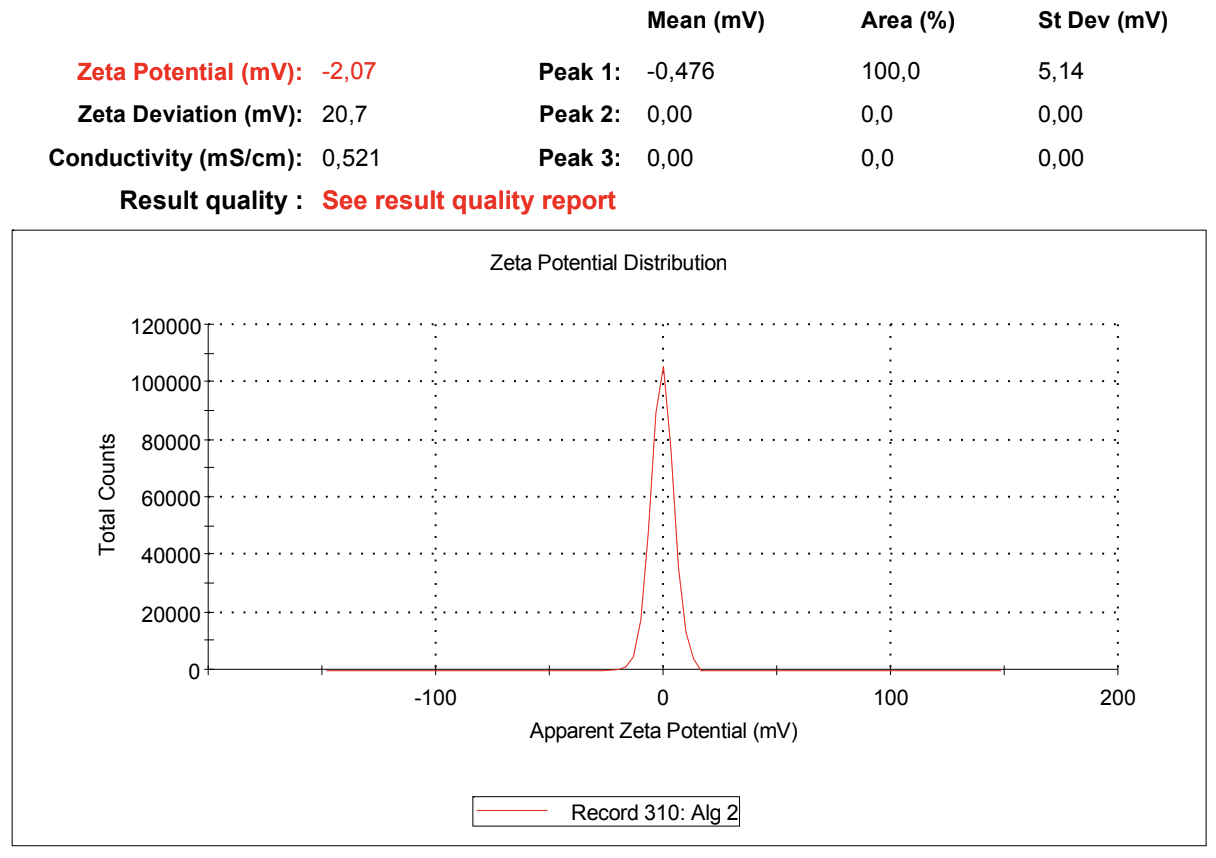

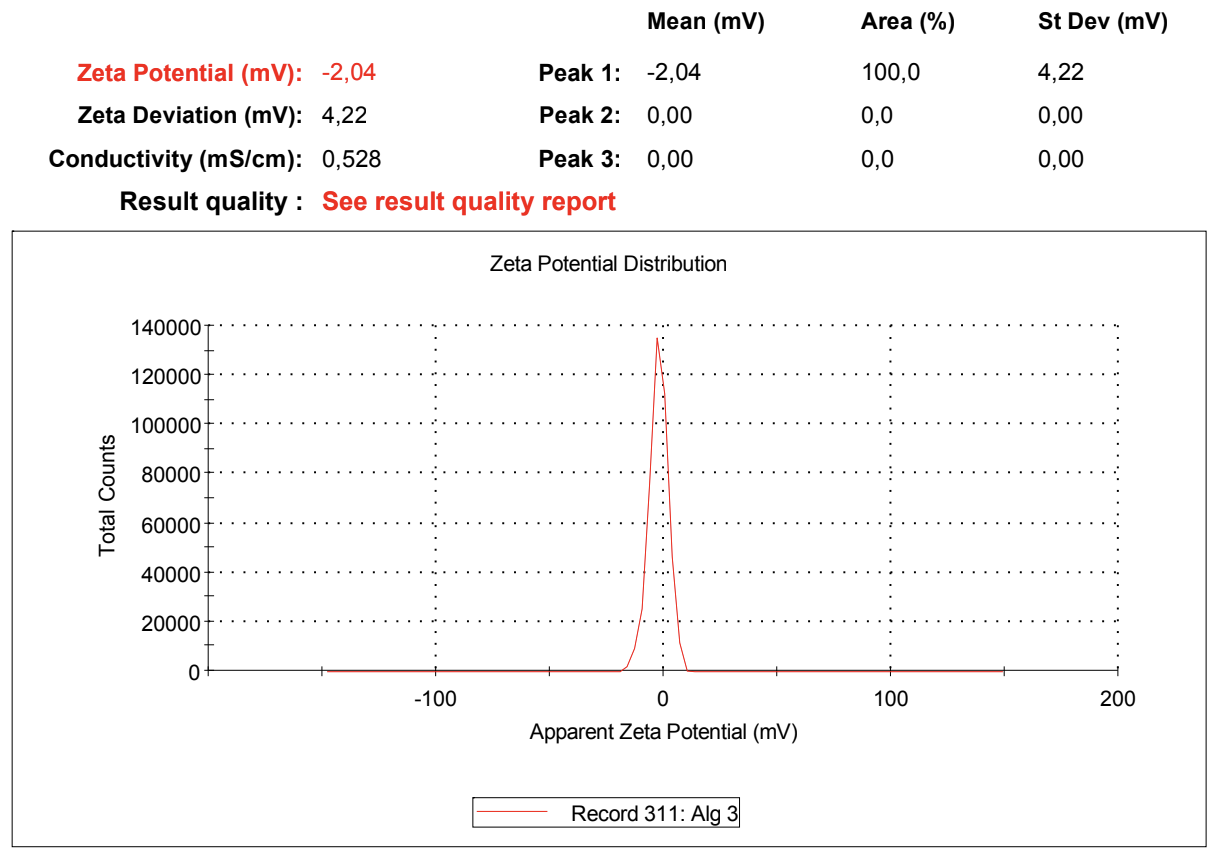


**Fig. S6.** Zeta potential analysis of Alg@FA-SeNPs showed values of -2.51, -2.07, and -2.04 mV, with an average surface charge of -2.21 mV, indicating a slightly negative surface potential likely due to the presence of alginate-CaCl_2_ on the nanoparticle surface.

**Zeta Potential of CS@FA-SeNPs**


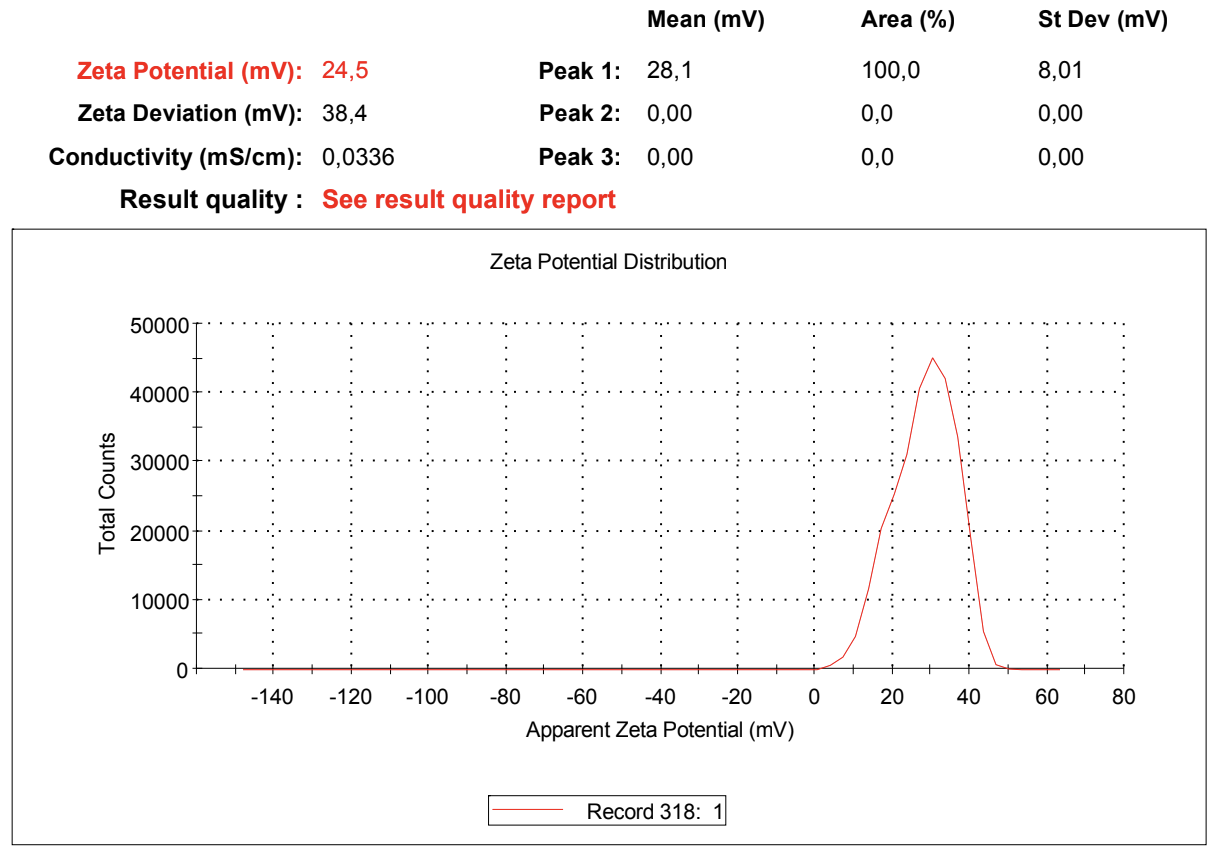

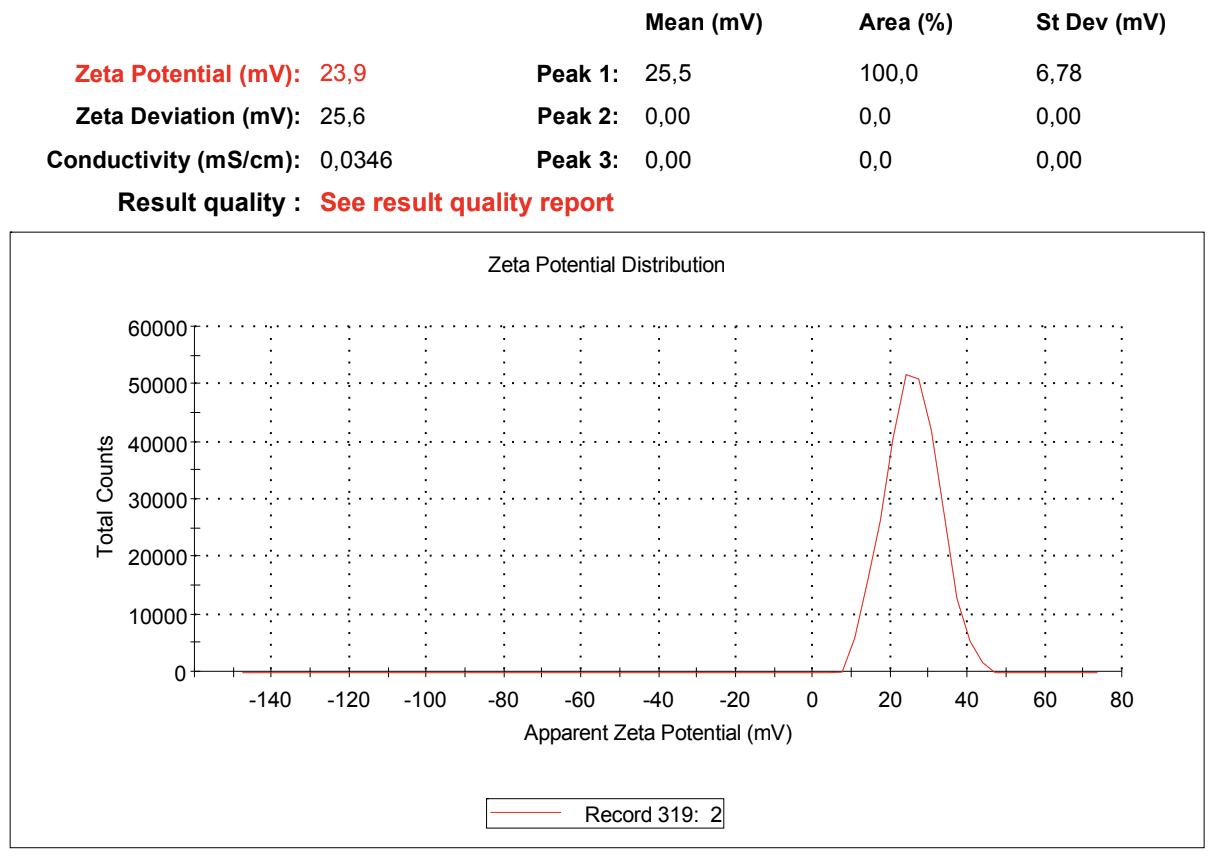

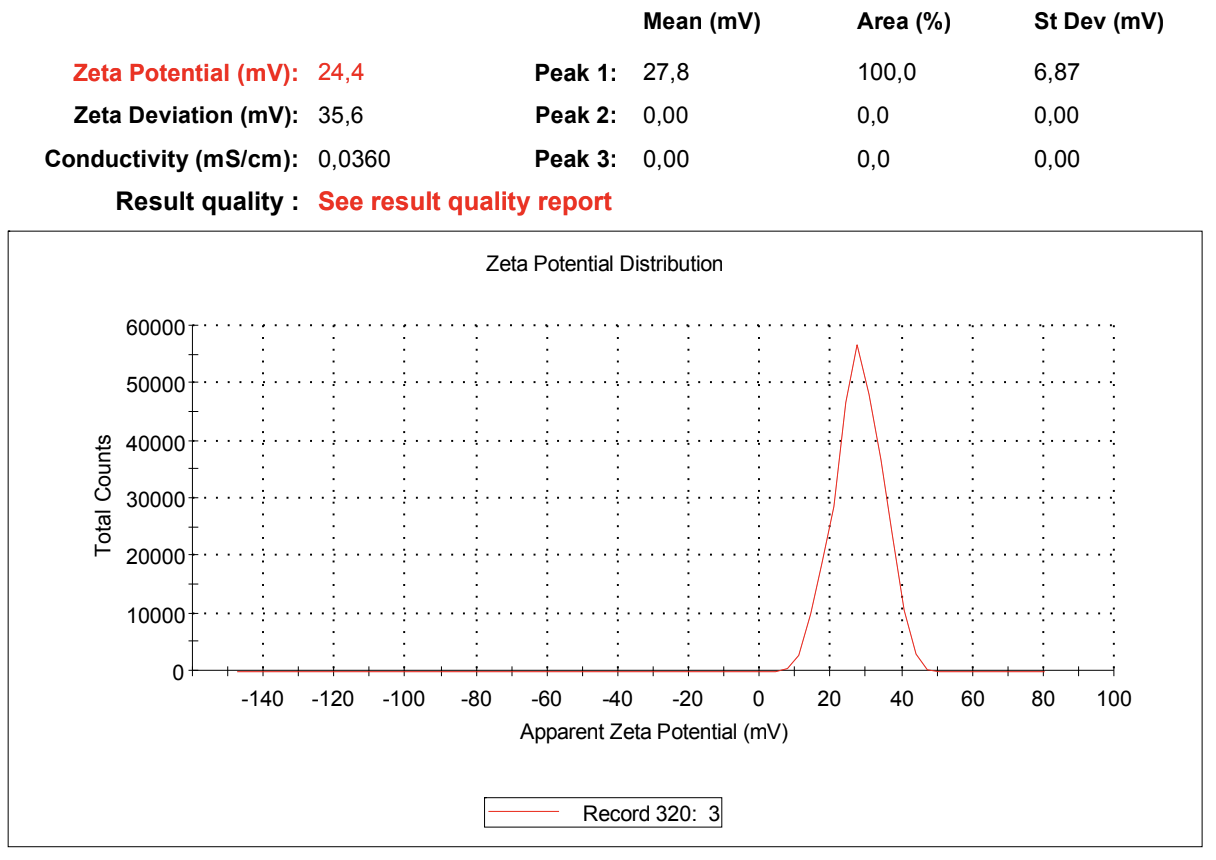


**Fig. S7** Zeta potential measurements of the CS@FA-SeNPs revealed values of 24.5, 23.9, and 24.4 mV, with an average surface charge of 24.3 mV, indicating good colloidal stability and a moderately positive surface charge.
